# A scalable platform for dumbbell siRNA production using engineered *Td* PIE in probiotic *E. coli*

**DOI:** 10.1016/j.omtn.2026.103030

**Published:** 2026-07-22

**Authors:** Zhibo Huang, Shaojiao Wang, Jinhao Liu, Yude Lin, Zhiwei Xiao, Tienan Chen, Bo Shi, Sijie Gu, Rujiang Zhou, Zhengju Yao, Xizhi Guo

**Affiliations:** 1 Bio-X Institutes, Key Laboratory for the Genetics of Developmental and Neuropsychiatric Disorders, Ministry of Education, Shanghai Jiao Tong University, Shanghai 200240, China

**Keywords:** MT: Non-coding RNAs, PIE, dumbbell-shaped siRNA, immunogenicity, probiotic, fermentation, RNA interference, permuted intron-exon, RNA circularization, Escherichia coli Nissle 1917, fed-batch fermentation, lipid nanoparticles, PCSK9

## Abstract

Despite the clinical success of small interfering RNA (siRNA) therapeutics, limitations including rapid degradation, off-target effects, and high manufacturing costs hinder broader application. Circular siRNAs, particularly dumbbell-shaped variants (db-siRNAs), offer enhanced stability and prolonged activity but lack scalable production methods. Here, we developed a streamlined platform for db-siRNA biosynthesis using a minimized T4 phage thymidylate synthase (*Td*) group I intron permuted intron-exon (PIE) system. By embedding essential splicing motifs into one terminal loop, we achieved ∼85% cyclization efficiency both *in vitro* and *in vivo*. Using engineered *Escherichia coli* Nissle 1917 (EcN), we established a low-immunogenicity production system, which could yield functional db-siRNAs at 60 mg/L in a fed-batch fermentation with a 3 L bioreactor. db-siRNAs with 23-bp stems demonstrated optimal gene silencing for over 10 days *in vitro* and effectively reduced target expression *in vivo* when formulated with lipid nanoparticles. Our platform enables rapid, cost-effective production of stable, potent db-siRNAs for preclinical development, addressing key manufacturing challenges in RNA interference (RNAi) therapeutics.

## Graphical abstract

**Figure:**
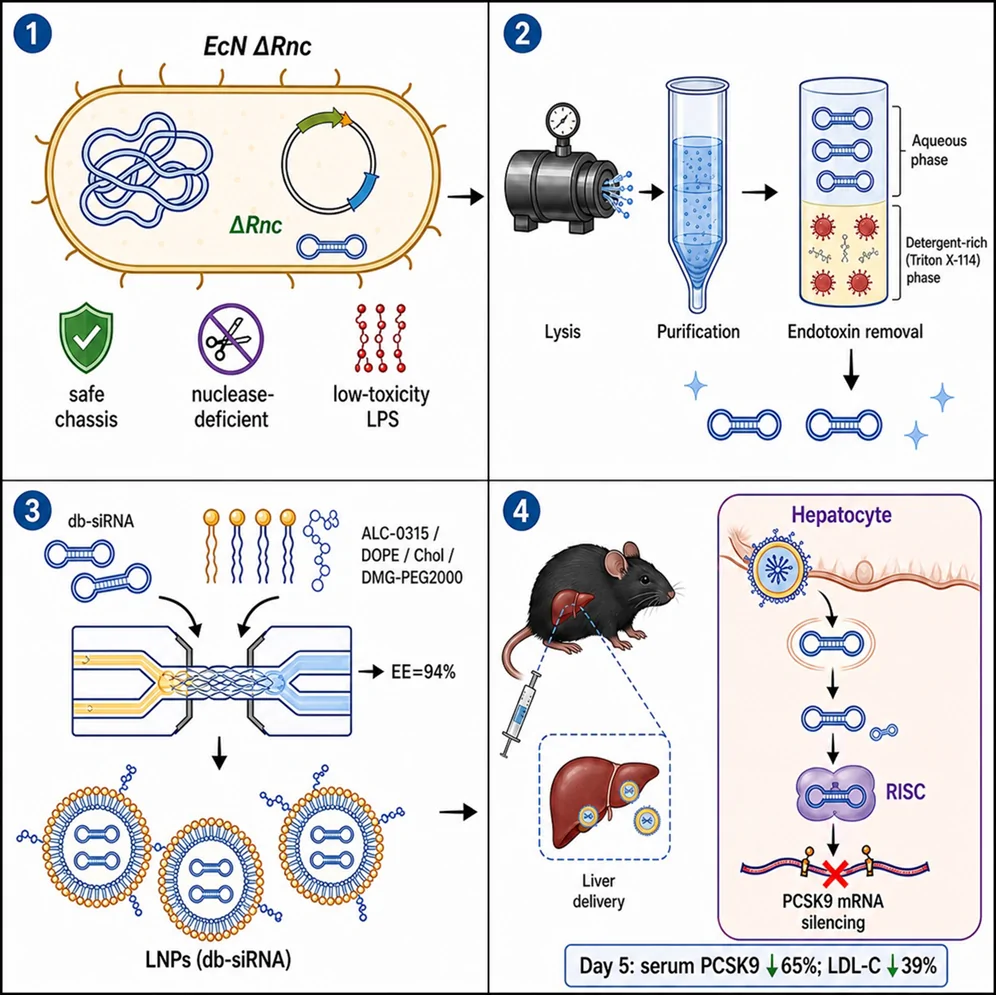

## Teaser

Guo and colleagues devised a streamlined, scalable platform for cost-effective production of potent db-siRNAs using engineered *Td* PIE in probiotic *E. coli*, addressing key manufacturing challenges in RNAi therapeutics for preclinical and clinical trials.

## Introduction

The discovery of RNA interference (RNAi) has marked a new era in the development of RNA-based drugs.^1^ Small interfering RNAs (siRNAs), typically 20–24 bp duplexes with 2-nt 3′ overhangs, are central to this pathway. After Dicer processing and RNA-induced silencing complex (RISC) loading, the antisense strand guides Ago2-mediated cleavage of complementary mRNAs in mammalian cells.^2^^,^^3^^,^^4^ To date, several siRNA drugs—including patisiran, givosiran, and inclisiran—have gained the US Food and Drug Administration (FDA) approval, highlighting their clinical potential.^5^ Additionally, numerous siRNA-based products are currently in various stages of clinical trials.

Despite progress, conventional linear siRNAs suffer from poor nuclease resistance and short half-lives in serum (∼5–10 min).^6^ This susceptibility to nucleases also poses challenges for the industrial production and storage of siRNAs, as well as their clinical applications. Another limitation is the potential for unintended “off-target” silencing of endogenous genes.^7^ To address these issues, a circular form of siRNAs has been developed to improve stability against serum and exonucleases and prolonged activity.^8^^,^^9^^,^^10^^,^^11^^,^^12^

Circular RNA (circRNA) is defined as a covalently closed non-coding RNA molecule generated by back-splicing in eukaryotic cells. In the context of RNA interference, circular duplex siRNAs have been engineered through two primary strategies: the self-annealing of a single chimeric oligonucleotide^11^ and the hybridization of a conventional guide strand with a circularized passenger strand.^9^ A distinct single-stranded form, referred to as caged siRNA, is constructed using a photocleavable linker to achieve complete circularization.^12^^,^^13^ A further optimized structure, the dumbbell siRNA (db-siRNA), is characterized by a central 21–23 bp duplex and terminal 7–9 nt loops. A key advantage of these circular architectures is their superior biostability; they exhibit increased exonuclease resistance, prolonged gene silencing activity, and minimized off-target effects relative to linear siRNAs in mammalian cells.^14^^,^^15^

Current db-siRNA production relies on chemical synthesis or enzymatic ligation, which are costly and difficult to scale. Ribozyme-based approaches, such as group I or II intron permutation of intronexon (PIE) system, enable autocatalytic RNA circularization.^16^^,^^17^^,^^18^ Traditional group I PIE method derived from the thymidylate synthase (*Td*) gene of the T4 phage or the pretRNA^Leu^ of *Anabaena* can add 74 to 186 extra nucleotides after self-splicing. More precise methods for producing scarless circRNAs without exonic nucleotides include the use of group I introns from pre-rRNA of *Tetrahymena*^17^ or group II introns from *Clostridium tetani* (*C. te*).^18^ To date, only a few group II introns have been capable of producing circRNAs by PIE in *Escherichia coli*. For example, a group II intron from *C. te* has been engineered to efficiently produce circRNAs of various sizes *in vivo*.^19^ The six domains (D1–D6) of the *C. te* group II intron are split and reconstituted to generate circRNAs in bacteria.^20^ However, the transcription of lengthy group II introns, which range from 700 to 900 nt, is energetically costly and yields low circularization efficiency for producing a small 64-nt circular siRNA.

Here, we report a streamlined, scalable platform for scarless db-siRNA biosynthesis using a minimized *Td* group I intron system. By embedding essential splicing motifs (E1/E2) into one terminal loop, we achieve high-efficiency self-splicing both *in vitro* and *in vivo*. Furthermore, we engineer the probiotic *E. coli* Nissle 1917 (EcN) to support low-immunogenicity, IPTG-inducible db-siRNA production. This platform enables rapid design, cost-effective manufacturing, and preclinical evaluation of gene-specific db-siRNAs.

## Results

### *In vitro* synthesis of db-siRNAs using an engineered *Td* PIE method

**Figure 1:**
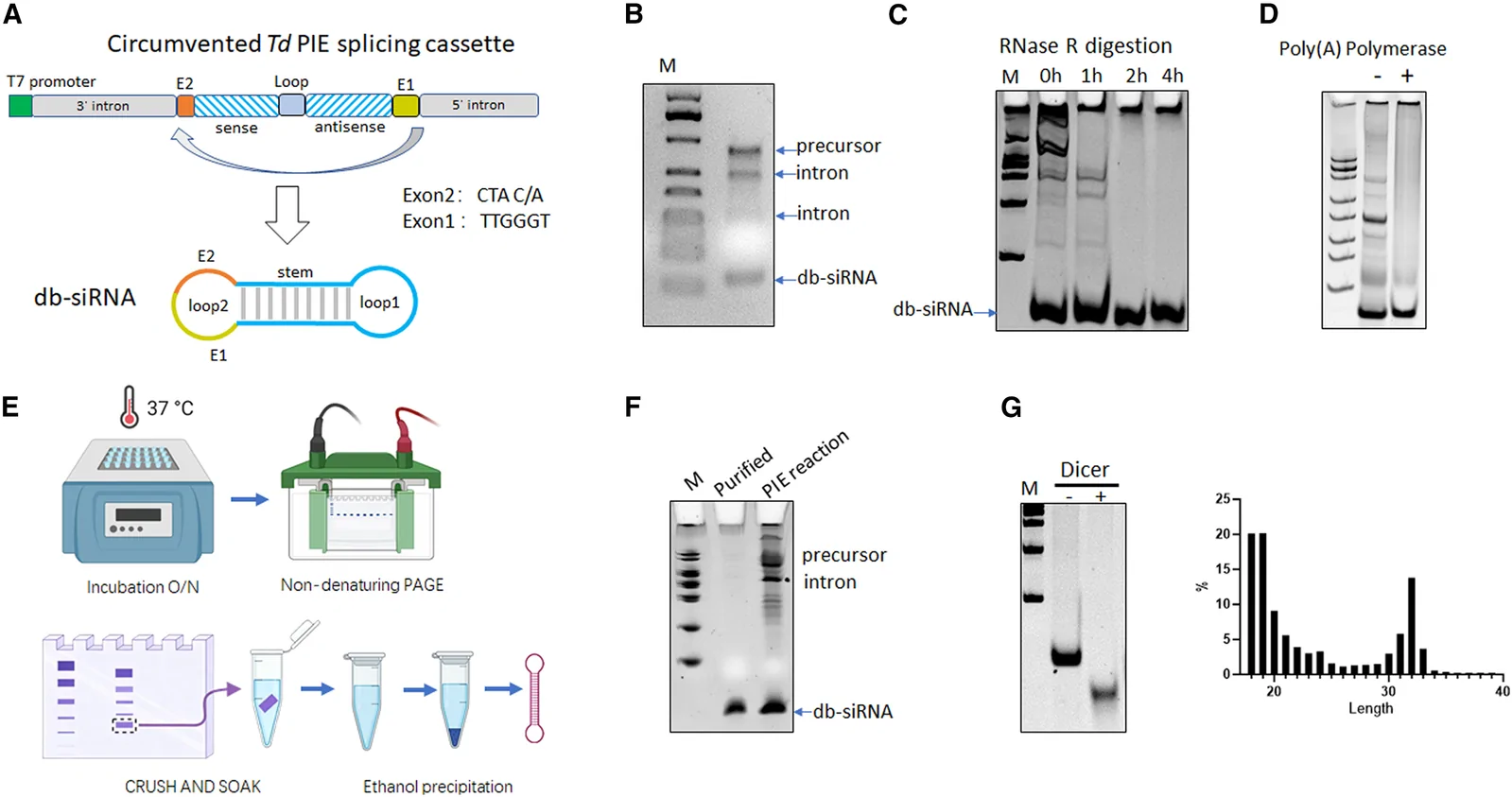
*In vitro* synthesis of dumbbell siRNA by the *Td* PIE method (A) Schematic depictions of the strategy of dumbbell siRNA synthesis by RNA self-splicing in an engineered group I *Td* PIE. The core exonic sequences for PIE splicing were minimized into E1 (TTGGGT) and E2 (CTAC/A). The DNA template that consisted of 3′ *Td* intron, a 65 nt siRNA backbone, and 5′ *Td* intron was *in vitro* transcription (IVT) by T7 RNA polymerase. Dumbbell siRNAs (db-siRNAs) that carried a 23-nt duplex and 9–10-nt closed loop at both sides were synthesized through RNA self-splicing. (B) Agarose gel electrophoresis for db-siRNA (siFluc6) post-*Td* PIE self-splicing. (C) Native PAGE gel electrophoresis for db-siRNAs after 1, 2, and 4 h of RNase R digestion. (D) Native PAGE gel electrophoresis for db-siRNAs with or without poly(A) polymerase incubation. (E) Diagram shows the purification process of db-siRNAs from PAGE gel slices by CRUSH and SOAK method. (F) Native PAGE gel electrophoresis for db-siRNAs with and without gel purification. (G) Native PAGE gel electrophoresis for db-siRNA after Dicer digestion. The relative abundance of RNA outputs at various size was calculated from RNA sequencing.

Mature db-siRNAs typically contain a central stem (21–23 bp) and two terminal loops (4–11 nt).^15^ The *Td*-group-I-intron-based PIE system requires minimal exonic recognition elements E1 (TTGGGT) and E2 (CTAC), which can be retained in the final product without impairing function.^21^ We designed a DNA template consisting of a 23-bp stem (sense and antisense strands), a 9-nt internal loop, and flanking E1 and E2 sequences (Figure 1A). This cassette was inserted between split 5′ and 3′ segments of the Td intron under a T7 promoter. Using siFluc6—a 23-nt guide targeting firefly luciferase (*Fluc*)—we performed *in vitro* transcription (IVT), followed by self-splicing at 37°C for 6 h. Agarose gel electrophoresis revealed efficient formation of a compact RNA band (Figure 1B). Native PAGE analysis further demonstrated that these db-siRNAs were resistant to RNase R digestion (Figure 1C) and polyA-polymerase-mediated elongation (Figure 1D), indicating the presence of fully closed terminal loops.

db-siRNAs were isolated from polyacrylamide gels (PAGE) slices using a CRUSH-and-SOAK method (Figure 1E). After overnight elution from the gel, db-siRNAs were precipitated using three volumes of cold ethanol with sufficient quality for subsequent experiments (Figure 1F). Dicer-mediated digestion of db-siRNAs predominantly contained 18–23 nt RNA (Figure 1G), as revealed by PAGE electrophoresis and RNA sequencing (RNA-seq). These results establish a robust workflow for IVT-based db-siRNA production.

To further confirm the sequence and circular topology of the db-siRNA product, RNA-seq was used to examine reads spanning the designed head-to-tail junction. Among 9,257,881 junction-containing reads, 9,117,497 reads perfectly matched the expected TTGGGT-CTAA junction, corresponding to a junction precision of 98.48% (Figure S1). Intact-mass analysis by MALDI-TOF MS detected a dominant molecular ion centered at approximately 20.56 kDa, very close to the theoretical molecular weight of the designed 65-nt db-siRNA (20.93 kDa). No prominent high-molecular-weight concatemers or low-molecular-weight degradation products were detected under the experimental conditions (Figure S2). Together with nuclease resistance and Dicer digestion analyses, these data support the structural identity and homogeneity of the purified db-siRNA product.

### Optimization of db-siRNA structure enhances silencing efficiency

**Figure 2:**
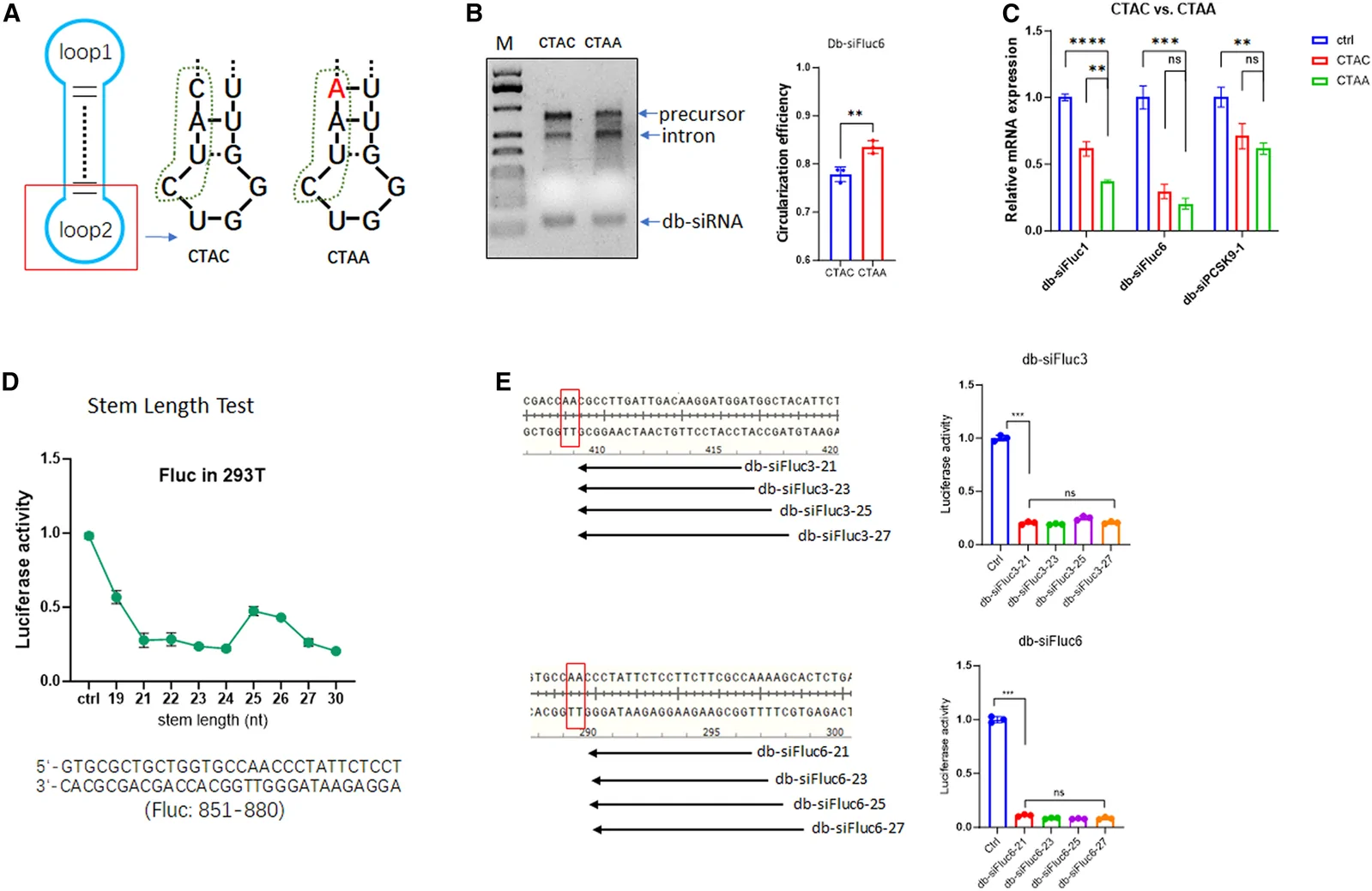
Optimization of db-siRNA design (A) A diagram depicts the variation of core E2 sequence (green dashed line, CTAC versus CTAA) in the loop cyclization of db-siRNAs. (B) Agarose gel electrophoresis for db-siRNAs (siFluc6) that was synthesized with E2 of CTAC or CTAA (left panel). Circularization efficiency was estimated by the gray density of precursor, intron, and db-siRNA bands (right panel). *n* = 3. (C) qPCR analysis for knockdown efficiency of various db-siRNAs targeted firefly luciferase (*Fluc*) or *PCSK9* gene in 293T cells. Ctrl, scramble siRNA control. *n* = 3. (D) Luciferase activity assay to compare knockdown efficiency of db-siRNAs with various duplex stem length ranging from 19 to 30 nt. Ctrl, scramble siRNA control. db-siRNAs targeted *Fluc* gene positions ranging from 851 to 880 bp. *n* = 3. (E) Luciferase activity assay to assess knockdown efficiency of db-siRNAs. siRNAs were designed to target *Fluc* gene at two positions (siFluc3 or 6) with duplex stem length ranging from 21 to 27 nt and extension sequence “TTGG”. Ctrl, scramble siRNA control. *n* = 3. Data are shown as mean ± SD; ∗, *p* < 0.05; ∗∗, *p* < 0.01; ∗∗∗, *p* < 0.001.

The 3′ terminal 1–2 nucleotides of the E2 sequence (CTAC), which are critical for *Td* PIE, can be altered without impairing circularization efficiency.^21^ We evaluated whether modifying the E2 terminus (CTAC versus CTAA) affects circularization or activity. This substitution potentially introduces an additional A–T base pair into the central stem region (indicated by green dashed lines and red letters in Figure 2A). The db-siRNA (db-siFluc6) that targets the *Fluc* gene was synthesized by PIE-method-based IVT RNA products. Based on densitometric analysis of precursor and intron bands in agarose gels, PIE method with the “CTAA” E2 terminus showed a circularization efficiency around 85% (Equation 1), which was slightly higher than those with the “CTAC” terminus (Figure 2B). To validate the advantage, three different db-siRNAs (db-siFluc1, db-siFluc6, and db-siPCSK9-1) were then synthesized using either the CTAC or CTAA E2 variants and transfected into 293T cells. The CTAA version induced more potent gene silencing than the CTAC version, as indicated by qPCR analysis (Figure 2C). Consequently, all subsequent db-siRNAs constructs used the CTAA-E2.

We next examined the impact of stem length on their gene-silencing efficacy. A series of db-siFluc RNAs targeting the *Fluc* gene (ranging position 851–880 bp) were synthesized with stem lengths ranging from 19 to 30 bp. When tested in *Fluc*-overexpressing 293T cells, db-siRNAs with stem lengths of 23, 24, or 30 bp exhibited the highest silencing activity, as evidenced by luciferase activity (Figure 2D). Given that part of the E1 (TTGGGT) may be processed into the mature siRNA after Dicer processing, we also assessed designs with a “TTGG” extension beyond the target site—potentially improving specificity.

Two sets of db-siRNAs (db-siFluc3 and db-siFluc6) targeting the *Fluc* gene positions with “TTGG” extension were synthesized with PIE method with stem lengths from 21 to 27 bp (indicated in left panels of Figure 2E). Transfection into 293T cells and relative luciferase assay revealed comparable knockdown efficiencies across all constructs (right panels, Figure 2E). Consistent with prior reports,^15^ db-siRNAs with a 23-bp stem region provided optimal balance of potency and safety. Thus, it was adopted in all subsequent db-siRNAs constructs.

To determine the optimal concentration for subsequent *in vitro* assays, db-siRNA was transfected using Lipo8000 at final concentrations of 0, 10, 20, 40, 60, and 80 nM, and gene silencing was assessed by a dual-luciferase reporter assay. Luciferase activity decreased in a dose-dependent manner and reached a maximal level at 40 nM. Increase of the db-siRNA concentration to 60 or 80 nM did not further enhance knockdown efficiency (Figure S3). Therefore, 40 nM was selected as the standard concentration for subsequent *in vitro* transfection experiments.

### db-siRNA intracellular localization and impact on cell viability

To evaluate intracellular localization post-delivery, Cy5-labeled db-siRNA was transfected into cells and analyzed by fluorescence microscopy. Cy5 fluorescence was broadly detected in the cytoplasmic and perinuclear regions (Figure S4). Confocal microscope observations revealed approximately 80% transfection efficiency of db-siRNA with Lipo8000.

The potential cytotoxicity of db-siRNA was also evaluated in HEK293T cells using CCK-8 assay 24 h post-transfection. Cells were treated with Lipo8000 alone or with Lipo8000-db-siRNA at 40, 80, and 120 nM. Cell viability remained above 90% in all db-siRNA-treated groups, and no significant reduction of cell number was observed between Lipo8000-db-siRNA and Lipo8000-only controls (Figure S5), indicating that db-siRNA did not induce obvious cytotoxicity.

### db-siRNAs mediate durable and potent gene silencing in mammalian cells

**Figure 3:**
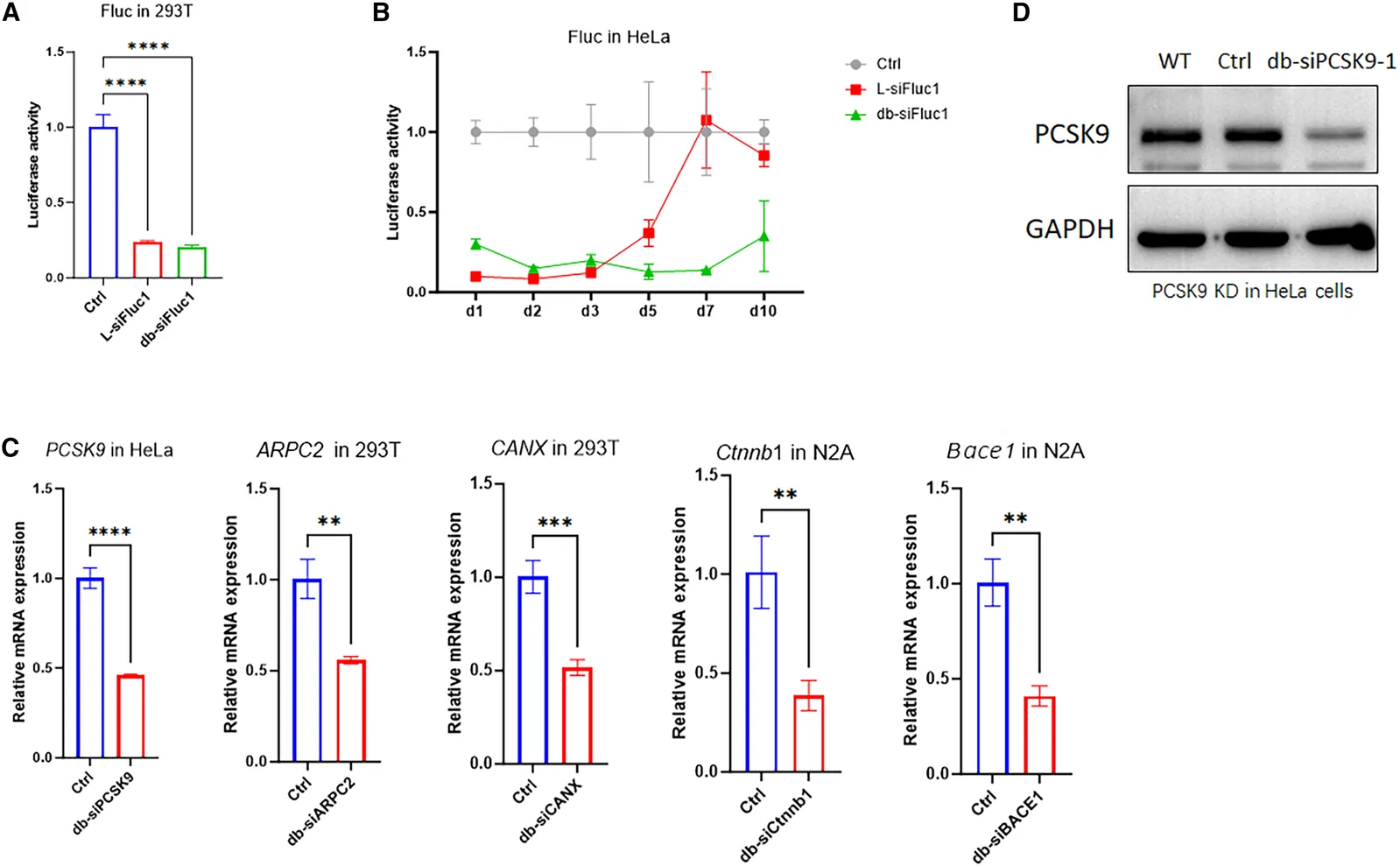
Gene silencing potency and duration of db-siRNAs (A) Luciferase activity assay to assess knockdown efficiency of db-siRNAs (db-siFluc1) and its linear counterpart (L-siFluc1) in 293T cells. *n* = 3. (B) Knockdown duration of db-siFluc1 and linear L-siFluc1 RNAs in 293T cells during 10-day cultures. (C) qPCR analysis for knockdown of endogenous gene expression by db-siRNAs in various cell lines. db-siRNAs were designed to target *PCSK9*, *ARPC2*, *CANX*, *Ctnnb1* and *Bace1* genes as indicated. *n* = 3. (D) Western blotting validated the knockdown of PCSK9 protein in HeLa cells by db-siPCSK9-1. Ctrl, scramble siRNA control. Data are shown as mean ± SD; ∗, *p* < 0.05; ∗∗, *p* < 0.01; ∗∗∗, *p* < 0.001.

We compared the gene-silencing efficacy of db-siRNAs (db-siFluc1) and their linear counterparts (L-siFluc1) in 293T cells. Db-siFluc1 silenced luciferase activity as effectively as linear L-siFluc1 (Figure 3A). However, in duration assays, db-siFluc1 suppressed luciferase activity for over 10 days, whereas L-siFluc1 lost efficacy within 7 days (Figure 3B). These results demonstrate the capacity of db-siRNAs to mediate long-term gene silencing.

We further tested db-siRNAs against endogenous genes: *PCSK9*, *ARPC2*, *CANX*, *Ctnnb1*, and *Bace1*. Transfection of the corresponding db-siRNAs (db-siPCSK9, db-siARPC2, db-siCANX, db-siCtnnb1, and db-siBace1) into HeLa, 293T, or Neuro2A cell lines significantly reduced target mRNA levels, as confirmed by qPCR (Figure 3C). Additionally, western blotting validated the suppression of PCSK9 protein expression by db-siPCSK9 (Figure 3D). These data demonstrate broad applicability and high potency of db-siRNAs across cell types and targets.

Serum stability was evaluated by incubating db-siRNA and linear siRNA in 20% fetal bovine serum at 37°C for 0, 1, 2, 4, and 24 h, followed by native PAGE analysis. Both RNA formats showed time-dependent degradation under this condition. The intact db-siRNA band decreased markedly and was barely detectable by 4 h, indicating that the current db-siRNA construct did not show improved extracellular serum stability compared with the linear siRNA control in this assay (Figure S6). This limitation suggests that further construct optimization or formulation protection may be required to improve serum stability.

### *In vivo* synthesis of db-siRNAs in *E. coli* strains

**Figure 4:**
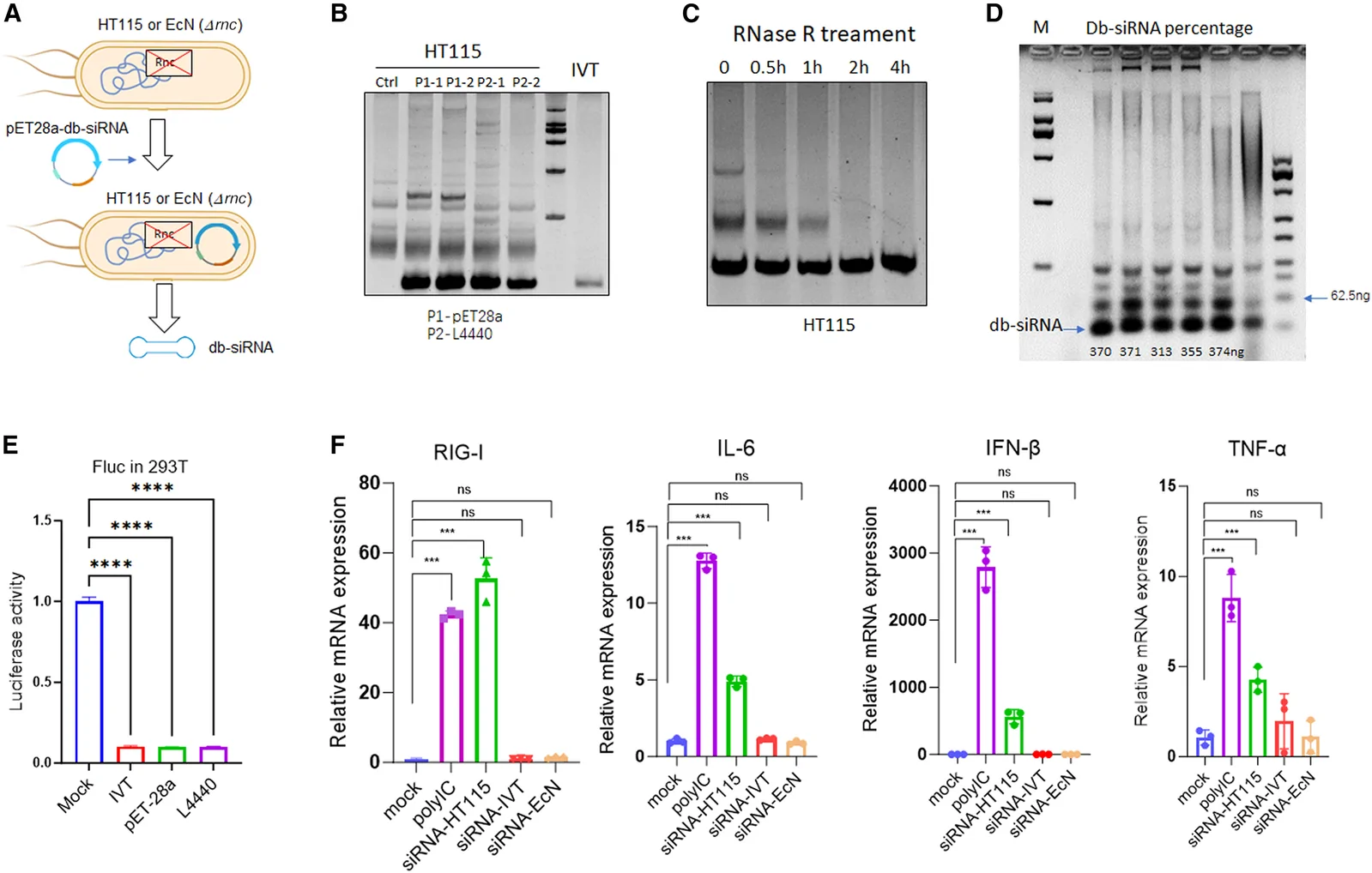
*In vivo* synthesis of db-siRNAs in *E. coli* (A) A diagram depicts the method of *in vivo* synthesis of db-siRNA in *E. coli* strains (HT115 or EcN) with *Rnc* gene deficiency. (B) Native PAGE gel electrophoresis for total RNAs that were transcribed in pET28a or L4440 vector in the HT115 strain. Ctrl, HT115 without transfection. (C) Native PAGE gel electrophoresis for db-siFluc6 RNAs isolated from the HT115 strain 0.5, 1, 2, and 4 h post-digestion by RNase R. (D) Agarose gel electrophoresis for total 2 μg RNA isolated from 6 individual EcN clones. The db-siRNA amount was evaluated by relative gray-density of bands as indicated. (E) Luciferase activity assay to assess knockdown efficiency of db-siFluc6 RNAs that were synthesized by IVT or pET28a and L4440 vectors in the HT115 strain. Db-siFluc6 were targeted to *Fluc* gene in 293T cells. Ctrl, scramble siRNA control. *n* = 3. (F) Immunogenicity of db-siFluc RNAs as evaluated by qPCR analysis for expression of *RIG-I*, *IL-6*, *IFN-β*, and *TNF-α* mRNAs in A549 cell lines. db-siFluc RNAs were synthesized by IVT or *in vivo* in HT115 and EcN strains and transfected A549 cells by Lipo8000. *n* = 3. Data are shown as mean ± SD; ∗, *p* < 0.05; ∗∗, *p* < 0.01; ∗∗∗, *p* < 0.001.

To enable scalable production, we cloned siFluc6 into pET28a- or L4440-based vectors and transformed them into RNase III (*rnc*)-deficient *E. coli* HT115 (Figure 4A). After induction with 0.1mg/mL IPTG overnight, db-siRNAs were efficiently synthesized from both vectors, as indicated by PAGE analysis (arrow in Figure 4B). These *in vivo*-synthesized db-siRNAs also exhibited resistance to RNase R digestion for up to 4 h (Figure 4C), indicating their structure integrity.

For clinical applicability of siRNAs, the immunogenicity of db-siRNA products needs to be assessed particularly considering potential contaminants from synthesis in bacteria. To eliminate residual endotoxins from HT115 during RNA induction and isolation, we also generated an *RNase III*-deficient probiotic *E. coli Nissle* 1917 (EcN) strain using CRISPR/Cas9.^22^ As a probiotic strain, the unique lipopolysaccharide (LPS) structure in EcN had no pyrogenic activity and has been proved to be safe in clinical investigation.^23^ When transformed with the pET28a-db-siFluc6 vector, EcN successfully produced db-siFluc6 that were resistant to RNase R digestion (Figures S7A and S7B). The yield of db-siRNA reached ∼17-18% total RNA in EcN (Figure 4D), which was comparable to that of HT115. Following purification via the CRUSH-and-SOAK method, db-siFluc6 derived from bacteria silenced luciferase activity as effectively as the IVT-synthesized siRNA (Figure 4E). These results establish a scalable method for *in vivo* db-siRNA production.

Purified db-siFluc6 RNAs from IVT, HT115, and EcN were transfected into A549 cells using Lipo8000. Although db-siRNAs from EcN contained a total endotoxin level comparable to that of HT115-derived products (Figure S7C), EcN-derived db-siRNAs induced markedly lower expression of retinoic-acid-inducible gene I (RIG-I), interleukin (IL)-6, interferon β (IFN-β), and tumor necrosis factor alpha (TNF-α) (Figure 4E). This reduced immunogenic response is consistent with the unique semi-rough LPS structure of EcN, which contains modified lipid A and shortened O-antigen chains and has substantially lower TLR4-activating activity than typical LPS from conventional laboratory *E. coli* strains.^24^^,^^25^ To further eliminate potential endotoxin effect, purified db-siRNA was subjected to Triton X-114 phase separation, and residual endotoxin was quantified by a recombinant factor C assay. This endotoxin removal step decreased endotoxin from approximately 0.65 EU/μg to approximately 0.0058 EU/μg after treatment (Figure S7D). These results indicate that the EcN chassis combined with endotoxin removal provides a low-immunogenic workflow for bacterial db-siRNA production.

### Large-scale production and purification of db-siRNA

**Figure 5:**
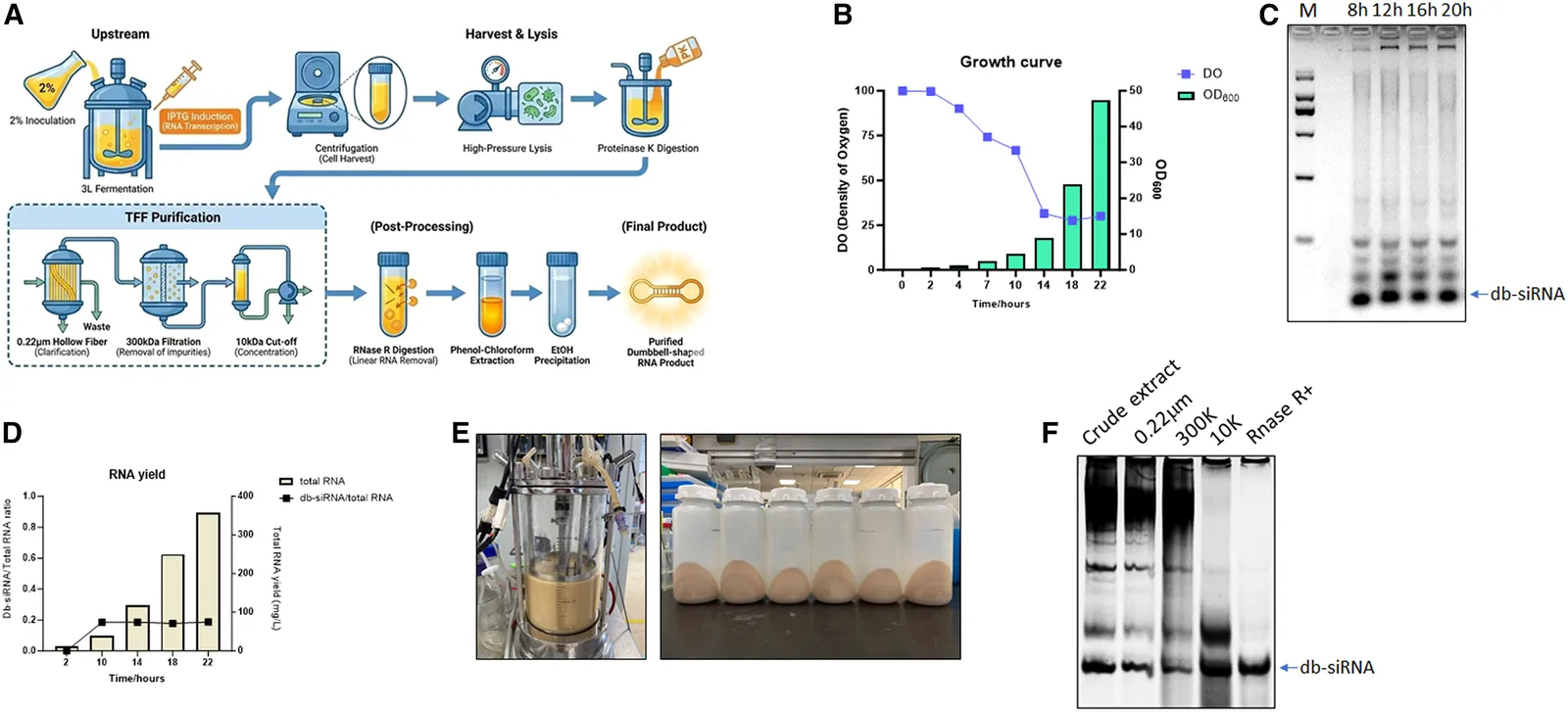
Large-scale production and purification of db-siRNA (A) Schematic workflow of large-scale db-siRNA production and purification. The process begins with fed-batch fermentation in a 3 L bioreactor, followed by cell harvest via centrifugation, high-pressure homogenization, proteinase K digestion, sequential tangential flow filtration (TFF), RNase R digestion, and finally phenol/chloroform extraction with ethanol precipitation. (B) Representative images of the culture after fermentation in a 3 L bioreactor and the harvested cells following centrifugation. (C) Growth curve of the EcN strain during overnight fermentation at 37°C. Cell density was monitored by measuring OD_600_ at the indicated time points after inoculation. Dissolved oxygen (DO) was maintained between 25% and 35%. (D) RNA yield was assessed by lysing samples collected at different time points after inoculation. The final total RNA yield reached up to 350 mg/L using fed-batch fermentation. (E) Agarose gel electrophoresis of RNA samples isolated at the indicated time points after inoculation. (F) PAGE analysis of RNA samples from successive TFF steps, including crude extract and samples retained by 0.22 μm, 300 kDa, and 10 kDa filters. The final product obtained after RNase R digestion and phenol/chloroform purification showed high purity.

To establish a scalable workflow for db-siRNA production, fed-batch fermentation was performed in a 3 L bioreactor (Figure 5A). An overnight culture of EcN in LB medium was expanded in 60 mL of Terrific Broth (TB) to an OD_600_ of ∼0.8, then inoculated into the bioreactor at 2% (v/v). When the OD_600_ reached 1.0–1.2, db-siRNA expression was induced by adding 0.5 mM IPTG. Fermentation was carried out at 37°C with dissolved oxygen (DO) maintained between 25% and 35%. Cells were harvested by centrifugation after approximately 22 h, when the OD_600_ reached a plateau around 45–50 (Figure 5B). Throughout the fermentation, db-siRNA synthesis remained at a high level (Figure 5C). From the 3 L bioreactor, nearly 180 g of wet cell mass was obtained, yielding approximately 1,050 mg of total RNA and ∼180 mg of db-siRNA (Figures 5D and 5E).

The harvested cells were lysed by three cycles of high-pressure homogenization. The supernatant was treated with proteinase K to remove proteins. Crude RNA was then purified by tangential flow filtration (TFF) using sequential hollow fiber columns with molecular weight cutoffs (MWCOs) of 0.22 μm (for cell debris removal), 300 kDa (to eliminate genomic DNA fragments), and 10 kDa (to retain and concentrate the RNA product). The resulting RNA was further digested with RNase R, followed by phenol/chloroform extraction and ethanol precipitation. The final db-siRNA product was obtained with high purity (Figure 5F).

### LNP-encapsulated db-siRNA silences gene expression in mice

**Figure 6:**
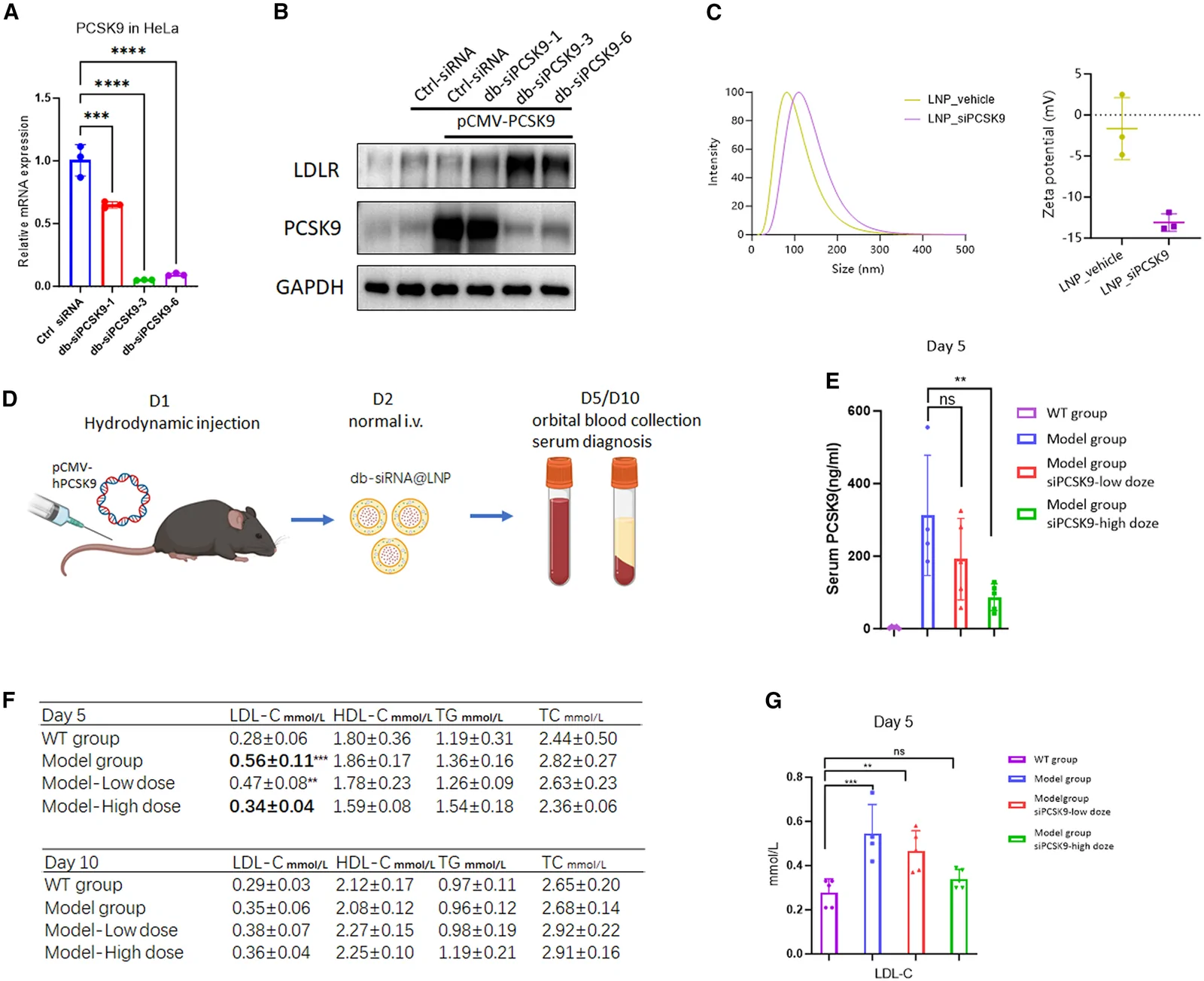
LNP-encapsulated db-siRNAs knockdown PCSK9 in mice (A) Assessment of db-siRNAs knockdown efficiency in HeLa cells by qPCR. Three db-siRNAs were designed to target PCSK9, indicated as db-siPCSK9-1/3/6. Ctrl, scramble siRNA control. *n* = 3. (B) Western blotting showed the decrease of PCSK9 and increase of LDLR protein expression in 293T cells by db-siPCSK9 RNAs. siCtrl, scramble siRNA control. (C) The average size and zeta potential of LNP-db-siRNAs mixtures were measured by NTA with triplicate. (D) Schematic depicting for the generation of hyperlipidemia mouse model by tail vein injection of pCMV-PCSK9 plasmids. (E) ELISA test for the PCSK9 levels in serum samples from hyperlipidemia mice treated with or without LNP-db-siPCSK9-3 RNAs. *n* = 5. (F and G) Statistics of LDL-C, HDL-C, TGs, and TC in serum samples from hyperlipidemia mice 5 or 10 days post-injection of LNP-db-siPCSK9-3 RNAs (F). At day 5, LDL-C was down-regulated by high-dose db-siRNAs in hyperlipidemia mice as compared to controls (G). *n* = 5. WT group, wild-type mice with injection of PBS buffer; model group, hyperlipidemia mice with PCSK9 plasmids injection; model-low dose, hyperlipidemia mice with low dose (5 μg/per mouse) injection of LNP-db-siRNAs; model-high dose, hyperlipidemia mice with high-dose (10 μg/per mouse) injection of LNP-db-siRNAs. LDL-C, low-density lipoprotein-cholesterol; HDL-C, high-density lipoprotein-cholesterol; TG, triglyceride; TC, total cholesterol. Data are shown as mean ± SD; ∗, *p* < 0.05; ∗∗, *p* < 0.01; ∗∗∗, *p* < 0.001.

For *in vivo* administration, LNPs were prepared via ethanol-based microfluidic mixing using ALC-0315:DOPE:cholesterol:DMG-PEG2000 at a molar ratio of 50:10:38.5:1.5 and an N/P ratio of 6. The resulting LNP-db-siRNA complexes had a concentration of 0.143 μg/μL, an average diameter of ∼100 nm, and a zeta potential of −13 mV (Figure 6C). With Quant-iT RiboGreen assay, both linear siRNA and db-siRNA achieved high encapsulation efficiencies of approximately 94% under the optimized N/P = 6 condition, indicating that the dumbbell topology did not compromise LNP encapsulation in this formulation (Figure S8A). LNP formulation has been reported to liver-specific trophism.^26^ With firefly luciferase mRNA encapsulated in LNPs and administered intravenously, *in vivo* imaging system (IVIS) showed that dose-dependent bioluminescence selectively enriched at the liver region, validating its hepatic target delivery and functional release (Figure S8B).

Inclisiran, an FDA-approved siRNA targeting *PCSK9*, achieves sustained lipid lowering with biannual dosing.^27^^,^^28^ Inspired by this, we designed three db-siPCSK9 candidates. Among them, db-siPCSK9-3 showed the strongest mRNA knockdown in HeLa cells (Figure 6A). Western blot analysis showed reduced PCSK9 protein levels and increased LDL receptor (LDLR) expression in 293T cells overexpressing pcDNA-PCSK9 (Figure 6B), making it suitable for *in vivo* delivery. A hyperlipidemic mouse model was then established by tail-vein injection of a human PCSK9-expressing plasmid (Figure 6D). Successful model induction was confirmed by ELISA detection of human PCSK9 in mouse serum (Figure 6E). Hyperlipidemic mice were divided into three groups: an untreated model group and two groups receiving low- (5 μg per mouse) or high-dose (10 μg per mouse) LNP-db-siPCSK9-3. On day 5 post-injection, the high-dose group showed significant reductions in both low-density lipoprotein-cholesterol (LDL-C) and hPCSK9 levels (Figures 6F and 6G), without affecting high-density lipoprotein-cholesterol (HDL-C), triglycerides (TGs), or total cholesterol (TC) levels (Figure S9). LDL-C levels in the model group rebounded to baseline by day 10, likely due to the transient nature of plasmid-mediated PCSK9 overexpression. The knockdown experiment demonstrated the potency of db-siRNA LNP encapsulation and *in vivo* efficacy.

## Discussion

Therapeutic siRNA development faces persistent challenges: instability in circulation, off-target effects, and high manufacturing costs. While chemical synthesis dominates clinical supply chains, it remains impractical for early-stage screening and preclinical studies. In this study, here, we present a novel, scalable biological platform for db-siRNA production based on the *Td* group I intron PIE system. This system allows convenient design of db-siRNAs against various genes by simply inserting a 23-nt antisense sequence into a template plasmid based on the pET28a vector. Our approach offers several advantages: (1) scarless design: by integrating E1/E2 into one terminal loop, we eliminate extraneous nucleotides; (2) high cyclization efficiency: ∼85%, far exceeding reported values for group II intron systems (<50%)^19^; (3) scalability: yields up to 60 mg/L in EcN within fermentation-scale production; (4) low immunogenicity: use of probiotic EcN minimizes endotoxin pyrogenic activity, reducing inflammatory risk; (5) stability: covalently closed structure confers resistance to RNases and long shelf life (>6 months at 4°C, data not shown). Collectively, our db-siRNA synthesis platform offers a robust tool for early-stage siRNA drug development, supporting long-term gene silencing both *in vitro* and *in vivo*.

Over the past decades, group I introns have been engineered to circularize RNAs with high efficiency in eukaryotic cells and *E. coli* strains such as BL21.^29^^,^^30^^,^^31^ However, conventional group-I-intron-mediated circularization often introduces exogenous sequences into the final RNA product, limiting its utility for circular siRNA synthesis. Several scarless PIE strategies have recently been developed to generate long circRNAs without extra nucleotides.^32^ For instance, a Clean-PIE system based on the *Td* group I intron was designed to produce protein-coding circRNAs by embedding E1/E2 sequences within the target open reading frame (ORF).^33^ Adopting a similar scarless strategy, we engineered the E1/E2 sequences to form a minimal 10-nt loop in the final db-siRNA structure after self-splicing. This streamlined *Td* intron PIE system efficiently produces db-siRNAs both *in vitro* and *in vivo*.

Compared to other ribozyme-based strategies, our system is simpler and more efficient. For instance, a PIE method derived from the *C*. *te* group II intron has also been employed to generate db-siRNAs without extra sequences in *E. coli* HT115. Indeed, *C. te* PIE-mediated circularization of db-siRNAs shows relatively low efficiency (less than 50%) compared to that of longer circRNAs.^19^ In contrast, our optimized *Td*-PIE-based system achieved a cyclization efficiency of approximately 85%. In line with the high circularization efficiency, our *Td*-PIE-based db-siRNA productivity reached ∼60 mg/L in EcN culture fermentation, which was much higher than that in *C. te* PIE system.^19^ The circular RNAs (40–100 nt) have also been produced in *E. coli* using hammerhead and hairpin ribozymes without introducing exogenous sequences.^34^ However, hammerhead-ribozyme-mediated production of 83–103 nt circRNAs achieved only 6.7%–9.5% cyclization efficiency. Given the compact and simple structure of *Td* intron arms, our strategy offers a practical and highly efficient solution for db-siRNA synthesis.

Consistent with previous studies,^15^ we found that db-siRNAs with stem lengths between 21 and 24 nt exhibited optimal gene knockdown. Interestingly, db-siRNAs with longer stems (27–30 nt) also showed substantial silencing activity (Figure 2D). Nevertheless, longer double-stranded RNAs (dsRNAs) are known to activate innate immune pathways, including RIG-I-like receptors and Toll-like receptors, leading to IFN responses.^35^ Observations from *in vitro* Dicer digestion assays show that db-siRNAs yield slightly shorter fragments (<20 nt). One strategy to mitigate this risk involves prioritizing antisense strands with downstream TT or TTGG extensions in design, which could optimize the balance between silencing efficacy, immunogenicity, and targeting accuracy. Therefore, to ensure safety *in vivo*, we recommend db-siRNAs with a 23-nt stem region.

To date, circRNAs and db-siRNAs have been predominantly produced in bacterial strains such as BL21 or HT115. Compared to BL21, the *RNase-III*-deficient HT115 strain supports more sustainable and high-yield db-siRNA synthesis. However, RNAs purified from HT115 cultures may be contaminated with endotoxins, which are abundant in the outer membrane of Gram-negative bacteria and can provoke innate immune responses,^36^ thereby compromising *in vivo* gene silencing. To circumvent endotoxin-related issues during RNA purification, we picked up the probiotic strain EcN as the host for db-siRNA production. The specific LPS structure in EcN cell membrane would not trigger pyrogenic activity.^23^^,^^25^ The lipid A of EcN carries specific phosphoethanolamine modification and distinct acylation patterns, which greatly weaken its binding affinity to the human TLR4/MD-2 complex, the core receptor complex responsible for sensing LPS and triggering pro-inflammatory signaling cascades.^25^ We used CRISPR/Cas9 to delete the *RNase III* gene (*rnc*) in the probiotic EcN strain. Although the db-siRNA product from EcN contained similarly high level of endotoxin (Figure S7C), its immunogenicity was quite low in cellular tests.

Furthermore, a fed-batch fermentation system using the EcN strain was optimized for large-scale db-siRNA production. A medium formulation consisting of K_2_HP O_4_/KH_2_PO_4_ buffer and glycerol as the carbon source supported high-density EcN growth, reaching an OD_600_ of approximately 40–50 in a 3 L bioreactor. Cells were harvested and lysed via high-pressure homogenization. Tangential flow filtration (TFF), employing sequential filtration through various hollow fiber columns, was used for db-siRNA purification. Notably, after collection and concentration using a 10 kDa hollow fiber column, the RNA product still contained residual rRNA fragments, which could be further eliminated by RNase R digestion. Nonetheless, there remains room to optimize TFF parameters—for instance, by reducing db-siRNA entrapment during early clearance steps with 0.22 μm and 300 kDa fiber columns and by incorporating additional hollow fiber columns with different MWCOs in intermediate TFF stages to enhance the purity of the final db-siRNA product.

In our *in vivo* administration test, the hydrodynamic plasmid model supports LNP-mediated delivery and functional *in vivo* knockdown for hPCSK9. The transient model was designed as a proof-of-concept test and therefore cannot conclusively evaluate db-siRNA long-term *in vivo* durability. It remains to be examined in more stable hyperlipidemic mouse models generating with AAV or hPCSK9 transgenic overexpression.

In summary, we have developed a versatile, scalable, and low-immunogenicity platform for db-siRNA biosynthesis based on *Td* PIE method. It enables rapid design and cost-effective production of stable, potent siRNAs for functional genomics and preclinical development. With further refinement, this system may accelerate the translation of RNAi therapeutics.

## Materials and methods

### Plasmid construction

DNA fragments encoding the T4 intron PIE, along with primers containing siRNA target sequences, were synthesized by Tsingke Biotechnology Co., Ltd. (Beijing, China). The T4 intron PIE cassette was inserted downstream of the T7 promoter in the backbone plasmids pET-28a(+) or L4440 using the Hieff Clone Universal One Step Cloning Kit (Yeasen, Cat# 10922), following the manufacturer’s instructions. The resulting plasmid was designated as the db-siRNA basal vector. Target-specific db-siRNA plasmids were constructed by introducing siRNA templates into the middle of the PIE cassette via homologous recombination. Each siRNA template consisted of an antisense strand, an internal loop, and a sense strand. All sequences and primers used are provided in Table S1.

### Generation of the EcN Δ*rnc* strain

The *rnc* gene was knocked out in the wild-type EcN strain using the pEcCas/pEcgRNA CRISPR-Cas9 system. Briefly, the plasmid pEcCas was first transformed into EcN. Arabinose was then added to the culture medium at a final concentration of 10 mM to induce expression of the λ-Red system. A 100 μL aliquot of competent cells was mixed with two guide RNA plasmids (pEgRNA-rnc1 and pEgRNA-rnc2, 100 ng each) and a donor DNA fragment (400 ng). The mixture was electroporated in a prechilled 2 mm Gene Pulser cuvette (Bio-Rad, Hercules, USA) at 2.5 kV. After electroporation, cells were recovered in LB medium for 1 h and then plated on LB agar containing both kanamycin and spectinomycin (50 μg/mL each). Putative *Δrnc* clones were verified by PCR and DNA sequencing, followed by curing of the CRISPR plasmids as previously described.^22^

### db-siRNA synthesis and purification

For *in vitro* siRNA synthesis, linear DNA templates containing the T7 promoter, PIE cassette, and target siRNA sequences were generated by PCR or restriction enzyme digestion. RNA transcription was performed using the Hi-yield T7 In Vitro Transcription Kit (N1-Me-pUTP) (HaiGene, Cat# H8P001SGS) according to the manufacturer’s protocol. Each 20 μL reaction contained 1 μg of linear DNA template; 2 μL of 10× Hi-Yield IVT Buffer A; 2 μL each of ATP, CTP, GTP, and UTP or N1-Me-pUTP (100 mM each); and 2 μL of Enzyme Mix. Reactions were incubated at 37°C for 6 h, followed by DNase I treatment to remove the DNA template.

For *in vivo* siRNA synthesis, the recombinant expression plasmid was transformed into competent *E. coli* HT115(DE3) or EcN *Δrnc* cells. Positive clones were inoculated into LB medium supplemented with kanamycin (50 μg/mL) and cultured at 37°C with shaking at 220 rpm until the OD_600_ reached approximately 0.6. Expression was induced with 0.1 mg/mL isopropyl β-d-1-thiogalactopyranoside (IPTG, Takara, Cat#9030) for 4 h (HT115) or 12 h (EcN *Δrnc*). Total RNA was extracted from cell pellets using TRIzol reagent (Vazyme, Cat# R401) according to the manufacturer’s instructions.

siRNA products were isolated and purified by electrophoresis on either a 10% native PAGE gel or a 2% agarose gel in 0.5× TBE buffer. Electrophoresis was performed at a constant voltage of 180 V for 50 min (native PAGE) or 40 min (agarose gel). Target siRNA bands were visualized under UV light and carefully excised with a clean razor blade. siRNAs were recovered from gel slices using the crush-and-soak method: gel pieces were crushed, and RNA was eluted with two volumes (v/w) of elution buffer (0.05 M NaAc, 0.1 mM EDTA) overnight at 4°C with gentle agitation. The supernatant was collected, and RNA was precipitated with 0.1 volume of 3 M NaAc (pH 5.2) and 3 volumes of ethanol at −20°C for 1 h. After high-speed centrifugation, RNA pellets were washed with 70% ethanol, air-dried, and dissolved in RNase-free water.

For quantification of RNA gel images, band intensities were measured using ImageJ software (National Institutes of Health). The circularization efficiency of Db-siRNA was calculated using Equation 1 :

Equation 1 $$\text{Circularization}\;\text{rate}=\frac{\text{Molecules}\_D\text{bsiRNA}\;}{\text{Molecules}\_D\text{bsiRNA}+\text{Molecules}\_\text{Linear}\;\text{Precursor}}\times 100\%$$

$$\text{Relative}\;\text{molecules}=\frac{\text{Intensity}\;}{\text{Molecular}\;\text{Weight}}$$

### db-siRNA validation and sequencing

To validate the circular structure of db-siRNAs, an RNase R digestion assay was conducted. Each reaction contained 5 μg of total RNA, 1 μL (20 U) of RNase R (Biori, Cat# BP-E08), and 2 μL of 10× RNase R buffer, made up to volume with RNase-free water. The mixture was incubated at 37°C, and 4 μL aliquots were collected at 0, 30, 60, 120, and 240 min. Each aliquot was combined with an equal volume of 2× RNA loading buffer and immediately flash-frozen in liquid nitrogen to terminate the reaction. The stability of circular siRNAs over the time course was assessed by electrophoresis on a 10% PAGE gel (180 V, 50 min).

To test the circularity of db-siRNA, RNA products were incubated with RNase R (#HBP004600, 1 U/μg RNA, 37°C, 2 h) or poly(A) polymerase (#R7070S, 1 U/2μg RNA, 37°C, 0.5 h), followed by electrophoresis.

To analyze mature siRNA duplexes generated from db-siRNA after Dicer digestion, 1 μg of purified db-siRNA was incubated with 0.1 μM recombinant human Dicer enzyme (IPODIX, Cat# Q9UPY3) at 37°C for 12 h in a reaction buffer containing 50 mM Tris-HCl (pH 7.5), 100 mM NaCl, 0.5 mM TCEP, and 2 mM MgCl_2_. Cleavage products were purified on a 15% PAGE gel, and correctly processed siRNA duplexes were recovered using the crush-and-soak method. These purified duplexes were subjected to high-throughput sequencing following a protocol similar to microRNA (miRNA) sequencing. Briefly, 3′ and 5′ adapters were sequentially ligated to the siRNAs, followed by reverse transcription and PCR amplification. The resulting cDNA libraries were sequenced on an Illumina platform to generate single-end reads. Raw sequencing data were processed by removing adapter sequences and low-quality bases using Cutadapt and FastQC. Clean reads were aligned to the original Db-siRNA sequences using a short-read aligner, and the frequency of cleavage events was analyzed to determine Dicer’s positional preference on db-siRNA substrates.

### RNA-seq junction mapping

Sequencing reads were processed to identify reads spanning the designed head-to-tail junction. Reads containing the terminal UUGGGU sequence and the 5′ head CUAA sequence were extracted and aligned to a linearized reference sequence containing the expected junction. Junction precision was calculated as the number of reads perfectly matching the expected UUGGGUCUAA junction divided by the total number of junction-containing reads. Oirignal sequence were uploaded at NCBI database (BioProject ID: PRJNA1505033, https://www.ncbi.nlm.nih.gov/bioproject/1505033).

### MALDI-TOF MS analysis of db-siRNA

The molecular mass of purified closed-circular db-siRNA was analyzed using a Shimadzu MALDI-7090 mass spectrometer in linear mode. Prior to analysis, RNA samples were desalted to reduce alkali metal adduct formation. The desalted RNA was mixed 1:1 (v/v) with matrix solution containing 3-hydroxypicolinic acid (3-HPA) or THAP supplemented with ammonium citrate, spotted onto a stainless-steel MALDI target, and air-dried using the dried-droplet method. Spectra were acquired with pulsed extraction set to 20,000, ion-gate blanking at m/z 500, laser power around 179, and laser diameter of 200. For each sample, 20 profiles were accumulated and processed with smoothing factor 200. External calibration was performed using a validated custom calibration method covering the 10–30 kDa mass range.

### Knockdown efficiency of db-siRNAs in cell lines

The RNAi efficiency of db-siRNAs was evaluated in 293T, HeLa, and Neuro2A cell lines, while the A549 cell line was used to assess immune responses. All cell lines (obtained from ATCC) were cultured in DMEM (Damas, Cat# C8274) supplemented with 100 μg/mL penicillin, 100 μg/mL streptomycin, and 10% fetal bovine serum (FBS) (Vazyme, Cat# F101-01) and maintained at 37°C in a humidified 5% CO_2_ incubator.

For RNAi efficacy assays, 293T or HeLa cells were seeded in 24-well plates one day before transfection to reach approximately 70% confluence. Transfection complexes were prepared using Lipo8000 transfection reagent (Beyotime, Cat# C0533) according to the manufacturer’s instructions. Unless otherwise indicated, cells were transfected with target-specific or scrambled siRNA at a final concentration of 40 nM. The scrambled negative-control siRNA was synthesized by Tsingke Biotechnology Co., Ltd. based on the sequence of Ambion Silencer Negative Control No. 1 siRNA (Thermo Fisher Scientific, AM4615). The sequences were as follows: sense strand, 5′-UUCUCCGAACGUGUCACGU(dT) (dT)-3′; antisense strand, 5′-ACGUGACACGUUCGGAGAA(dT) (dT)-3′.

The potential immunogenicity of db-siRNA was evaluated in A549 cells. Cells at approximately 70% confluence were transfected with 400 ng of db-siRNA synthesized by IVT, or produced in *E. coli* HT115(DE3) or *E. coli* EcN *ΔRnc*, using Lipo8000 reagent. Positive control cells were treated with 400 ng of poly(I:C), while the negative control received transfection reagent only. Twenty-four hours post-transfection, immunogenicity was assessed by measuring the mRNA expression levels of RIG-I, IL-6, IFN-β, and TNF-α via qPCR.

HEK293T or HeLa cells were seeded in 24-well plates and grown to approximately 70% confluence before transfection. For each well, cells were co-transfected with 20 ng of firefly luciferase reporter plasmid pGL3-Control (Promega), 20 ng of pRL-TK internal control plasmid (Promega), and db-siRNA or scrambled siRNA using 0.8 μL of Lipo8000 transfection reagent according to the manufacturer’s protocol. For concentration optimization, db-siRNA was tested at final concentrations of 0, 10, 20, 40, 60, and 80 nM. For subsequent luciferase assays, 40 nM db-siRNA was used unless otherwise indicated. Luciferase activity was measured 24 h after transfection using the Dual-Luciferase Reporter Assay System. Firefly luciferase signals were normalized to Renilla luciferase signals, and relative luminescence units were compared with the scrambled control group to determine gene-silencing efficiency.

For long-term RNAi activity assessment, HeLa, or 293T cells were seeded at an initial density of 50% confluence. After siRNA transfection, the culture medium was refreshed every 2 to 3 days, and luciferase activity was measured on days 1, 2, 3, 5, and 10 post-transfection.

### Fluorescence microscopy of Cy5-labeled db-siRNA uptake

Cells were seeded on glass-bottom dishes or coverslips and transfected with Cy5-labeled db-siRNA using Lipo8000 transfection reagent. At 24 h after transfection, cells were washed three times with PBS to remove extracellular fluorescence signal and fixed with 4% paraformaldehyde for 15 min at room temperature. Nuclei were stained with DAPI for 5 min, followed by three washes with PBS. Fluorescence images were acquired using a fluorescence or confocal microscope with DAPI and Cy5 channels. Intracellular Cy5 fluorescence was used to assess cellular uptake and subcellular localization of db-siRNA.

### Cell viability assay

The cytotoxicity of db-siRNA was evaluated using a CCK-8 assay (YEASEN, #40203ES76). HEK293T cells were seeded in 96-well plates and transfected with db-siRNA at final concentrations of 40, 80, or 120 nM using Lipo8000 transfection reagent. Untreated cells and Lipo8000-only-treated cells were included as controls. After 24 h of incubation, CCK-8 reagent was added to each well, and cells were further incubated according to the manufacturer’s protocol. Absorbance at 450 nm was measured using a microplate reader. Cell viability was calculated by normalization to untreated cells.

### Serum stability assay

For serum stability analysis, db-siRNA or linear siRNA was incubated in medium containing 20% FBS at 37°C. Samples were collected at 0, 1, 2, 4, and 24 h, immediately mixed with loading buffer, and stored on ice until electrophoresis. RNA integrity was analyzed by native PAGE, followed by gel staining and imaging.

### qPCR

Total RNA was extracted using TRIzol reagent 24 to 48 h after transfection. cDNA was synthesized using the Hiscript III reverse transcription system (Vazyme, Cat# R223). Quantitative PCR was performed on a Roche 480 real-time PCR system using SYBR Green (Vazyme, Cat# Q711). The expression levels of the following target genes were analyzed: ARPC2 and CANX in 293T cells, PCSK9 in HeLa cells, and Ctnnb1 and BACE1 in Neuro2A cells. Primer sequences are listed in Table S1.

### Western blotting

Cells were lysed with T-PER lysis buffer (Thermo Fisher Scientific, Cat# 78510) for 15 min at 4°C. Protein samples were separated by SDS-PAGE, transferred to an Immobilon-PVDF membrane (Millipore, Cat# IPVH00010), and blocked with 5% non-fat milk. The membranes were incubated overnight at 4°C with primary antibodies against PCSK9 (1:1,000; Abcam, Cat# ab181142), LDLR (1:1,000; Abclonal, Cat# A20808), and GAPDH (1:8,000; Proteintech, Cat# 60004-1-Ig). Proteins were visualized using horseradish peroxidase (HRP)-conjugated secondary antibodies and a chemiluminescent HRP substrate.

### Fed-batch fermentation in 3 L bioreactor

Large-scale production of db-siRNA was carried out in a 3 L bioreactor using a fed-batch fermentation strategy. EcN seed cultures were prepared by overnight growth in LB medium and subsequently expanded in 60 mL Terrific Broth (TB) to an OD_600_ of ∼0.8 before inoculation into the bioreactor at 2% (v/v). The basal medium (total volume 2 L) consisted of two separately sterilized components to avoid precipitation. Group A contained K_2_HPO_4_ (25 g), KH_2_PO_4_ (7.6 g), and glycerol (20 g) in 200 mL H_2_O. Group B contained MgSO_4_·7H_2_O (1.2 g), NH_4_Cl (8 g), tryptone (24 g), and yeast extract (48 g) in 1,800 mL H_2_O. Both components were sterilized at 121°C for 15 min and combined prior to fermentation.

Fermentation was conducted at 37°C with pH control using 1 M NH4OH or HCl. Dissolved oxygen (DO) was maintained at 25%–35% by cascading agitation between 200 and 1,000 rpm. Upon depletion of the initial carbon source, as indicated by a rise in pH, a concentrated feed medium (1 L) containing glycerol (300 g), yeast extract (40 g), and MgSO_4_·7H_2_O (0.3 g), sterilized at 121°C for 15 min, was supplied at a constant rate of 20 mL/h. Cell growth was monitored by OD_600_ measurements.

### TFF-based RNA purification for large-scale fermentation

Cells were harvested by centrifugation (6,000 × g, 15 min, 4°C), resuspended in PBS (1:10, w/v), and disrupted by high-pressure homogenization (800–1,000 bar, three cycles). The crude lysate was treated with proteinase K (50 μg/mL, 37°C, 1 h) to reduce viscosity and improve filtration performance.

Purification was performed using a sequential tangential flow filtration (TFF) process. The lysate was first clarified using a 0.22 μm membrane to remove insoluble debris. The permeate was then fractionated using a 300 kDa MWCO membrane, retaining large nucleic acids while allowing db-siRNA and other small RNAs to pass. The 300 kDa permeate was subsequently concentrated and diafiltered using a 10 kDa MWCO membrane with six volumes of buffer.

The concentrated RNA fraction was treated with RNase R (1 U/μg RNA, 37°C, 1 h) to selectively remove residual linear RNAs. Final purification was achieved by phenol-chloroform extraction and ethanol precipitation, and RNA concentration and purity were analyzed by NanoDrop and PAGE.

### Endotoxin removal and residual endotoxin quantification

Purified RNA samples were treated with Triton X-114 at a final concentration of 1% (v/v). The mixture was gently mixed and incubated on ice for 5–10 min to allow interaction between Triton X-114 and endotoxin. Samples were then incubated at 37°C for 10–15 min to induce phase separation and centrifuged at 12,000 × g for 5 min at 37°C. The upper aqueous phase containing RNA was carefully transferred to a new RNase-free and endotoxin-free tube, while the lower detergent-rich phase and interfacial layer were discarded. The phase-separation step was repeated when necessary. RNA was recovered from the aqueous phase and used for subsequent analysis.

Residual endotoxin levels in RNA samples were quantified using a recombinant factor C (rFC) endotoxin assay kit (RHINO BIO, RAF-02) according to the manufacturer’s instructions. RNA samples were diluted in endotoxin-free water to fall within the linear range of the standard curve. Fluorescence signals were measured using a microplate reader, and endotoxin levels were calculated from the standard curve and normalized to RNA mass as EU/μg RNA.

### LNP encapsulation of db-siRNAs

db-siRNAs were encapsulated into LNPs using a standard formulation consisting of ALC-0315 [AVT (Shanghai) Pharmaceutical Tech Co., Ltd., Cat# O02008], DOPE (AVT, Cat# S03005), cholesterol (AVT, Cat# O01001), and DMG-PEG2000 (AVT, Cat# O02005) at a molar ratio of 50:10:38.5:1.5. LNPs were assembled using a microfluidic system [NWDPS II 40, Nanowetech (Guangzhou) Instrument Co., Ltd.]. The lipid components dissolved in ethanol and the db-siRNA dissolved in citrate buffer (pH 4.0) were simultaneously injected into a microfluidic chip at an aqueous-to-organic phase flow rate ratio of 3:1 and an N/P ratio of 6. The resulting LNP solution was immediately diluted 30-fold with 1× PBS and concentrated by centrifugation at 3,000 × g for 40 min at 4°C using 10 kDa MWCO Amicon Ultra centrifugal filters. This concentration step was repeated, and the final LNP pellets were resuspended in 1× PBS to the desired volume. The hydrodynamic diameter, polydispersity index (PDI), and zeta potential of the purified LNPs were measured using a NanoBrook Omni particle size and zeta potential analyzer (Brookhaven Instruments).

### LNP encapsulation efficiency evaluation

RNA encapsulation efficiency was measured using the Quant-iT RiboGreen RNA Assay Kit. Freshly prepared LNP samples were diluted in assay buffer. Fluorescence from unencapsulated RNA was measured directly without detergent treatment. Total RNA fluorescence was measured after disrupting LNPs with Triton X-100 at a final concentration of 0.5%. Encapsulation efficiency was calculated using the following equation: EE (%) = [1− (F_unencapsulated/F_total)] × 100%, where F_unencapsulated represents fluorescence measured without Triton X-100 and F_total represents fluorescence measured after Triton X-100 lysis.

### Calculation of RNA loading efficiency

RNA loading efficiency was calculated from the lipid-to-RNA mass ratio derived from the formulation composition and N/P ratio. The mass fraction of RNA in LNPs was determined as RNA loading efficiency (%) = (m_RNA × EE)/(m_RNA × EE + m_lipid) × 100%, where m_RNA is the input RNA mass, EE is the encapsulation efficiency, and m_lipid is the total lipid mass. For the ALC-0315/DOPE/cholesterol/DMG-PEG2000 formulation (50:10:38.5:1.5, mol/mol) prepared at N/P = 6, the loading efficiency was calculated using the molecular weight of the RNA and the average molecular weight of the lipid mixture.

### *In vivo* hepatic delivery validation using mRNA-LNPs

To validate hepatic delivery of the LNP formulation, firefly luciferase mRNA was encapsulated into LNPs using the same microfluidic mixing procedure. Mice were intravenously injected with 0, 5, or 10 μg LNP-Fluc mRNA. At 12 h after injection, mice were administered D-luciferin (ShareBio, #SB-D119) and imaged using an IVIS Spectrum imaging system. Bioluminescence signals were used to evaluate *in vivo* delivery and functional expression of the LNP cargo.

### *In vivo* silencing of db-siRNAs in a hyperlipidemia mouse model

Male C57BL/6 mice (6–8 weeks old) were obtained from Qinglongshan Experimental Animal Breeding Farm (Nanjing, Jiangsu, China) and housed under specific pathogen-free (SPF) conditions with a 12 h light/dark cycle, temperature maintained at 22 ± 2°C, humidity at 50% ± 10%, and free access to food and water. All animal experiments were conducted by protocol no. A2025108 in accordance with the ethical guidelines of Shanghai Jiao Tong University (approval no. SYXK 2023-0042).

After one week of acclimation, a hyperlipidemic mouse model was established on day 0 (D0) by a single rapid (5–7 s) intravenous injection via the tail vein of 2 mL saline containing 50 μg of pCMV-hPCSK9 plasmid. Control mice received an equal volume of saline alone. On day 1 (D1) post-modeling, mice were divided into groups and administered LNP-formulated siRNA therapeutics at low or high doses via intravenous injection. Blood samples were collected from the retro-orbital plexus on days 5 (D5) and 10 (D10) after LNP injection. Serum was separated by centrifugation and stored at −80°C for subsequent analysis.

The concentration of human PCSK9 (hPCSK9) in mouse serum was quantified using a commercial human proprotein convertase 9/PCSK9 ELISA Kit (LiankeBio, Cat# EK1124) according to the manufacturer’s instructions. TC, TGs, HDL-C, and LDL-C levels were measured using an automated biochemical analyzer (Cobas c311, Roche).

### Statistical analysis

All statistical analyses were performed using GraphPad Prism software (v.10). Data are presented as mean ± standard deviation (SD). Comparisons between two groups were analyzed using an unpaired Student’s *t* test. For comparisons among more than two groups, one-way analysis of variance (ANOVA) was applied, followed by an appropriate post hoc test for multiple comparisons. A *p* value of less than 0.05 was considered statistically significant.

## Data and code availability

All data are available in the main text or supplementary materials. Plasmids and bacterial strains will be made available upon request through Material Transfer Agreement.

## Acknowledgments

We thank members of the Guo laboratory for technical assistance and discussions. This work was supported by research grants from the 10.13039/501100012166National Key R&D Program of China (973 Program) (2020YFA0803601) and 10.13039/100014718National Natural Science Foundation of China (92068203 and 91749103) to X.G. and the open funding of Aging Mechanisms and Interventions Key Laboratory of Sichuan Province (SYS23-01) to T.C.

## Author contributions

Z.H., S.W., J.L., Y.L., and Z.X. performed experiments; X.G. designed experiments and supported investigations; T.C., B.S., S.G., R.Z., and Z.Y. helped preparing samples. X.G. wrote the manuscript.

## Declaration of interests

The *Td*-PIE-mediated db-siRNA synthesis and purification method has been patented by the authors.

## Supplemental information

Document S1. Figures S1–S9

Table S1. The DNA sequences of siRNA, oligos for PCR, and EcgRNA for genome editing were listed in Table S1 as an Excel file

Document S2. Article plus supplemental information
