# Supplementary material for: A scalable platform for dumbbell siRNA production using engineered Td PIE in probiotic E. coli
Source: Mol Ther Nucleic Acids. 2026 Jul 22;37(3):103030. doi: 10.1016/j.omtn.2026.103030 (PMC13487835; doi:10.1016/j.omtn.2026.103030)
Supplement: Supplementary file 1 [file mmc1.pdf]

OMTN, Volume *37*

Supplemental information

A scalable platform for dumbbell siRNA production using engineered *Td* PIE in probiotic *E. coli*

Zhibo Huang, Shaojiao Wang, Jinhao Liu, Yude Lin, Zhiwei Xiao, Tienan Chen, Bo Shi, Sijie Gu, Rujiang Zhou, Zhengju Yao, and Xizhi Guo

Supplemental figures

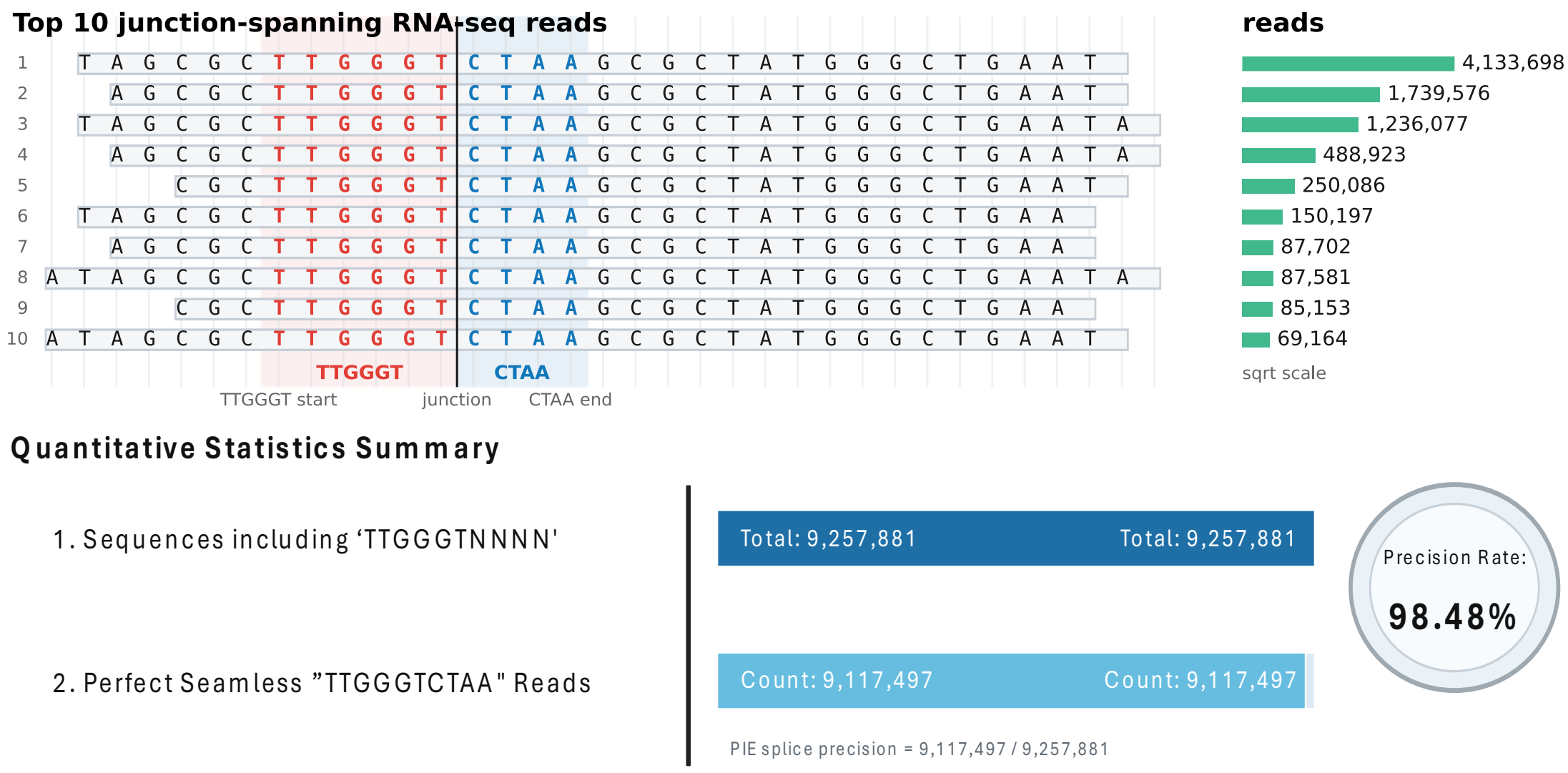

Figure S1. RNA-seq junction mapping of db-siRNA.

Junction-spanning reads containing the terminal TTGGGT sequence and the 5′ head CTAA sequence were aligned to a linearized junction reference. The expected TTGGGT-CTAA junction accounted for 98.48% of junction-containing reads.

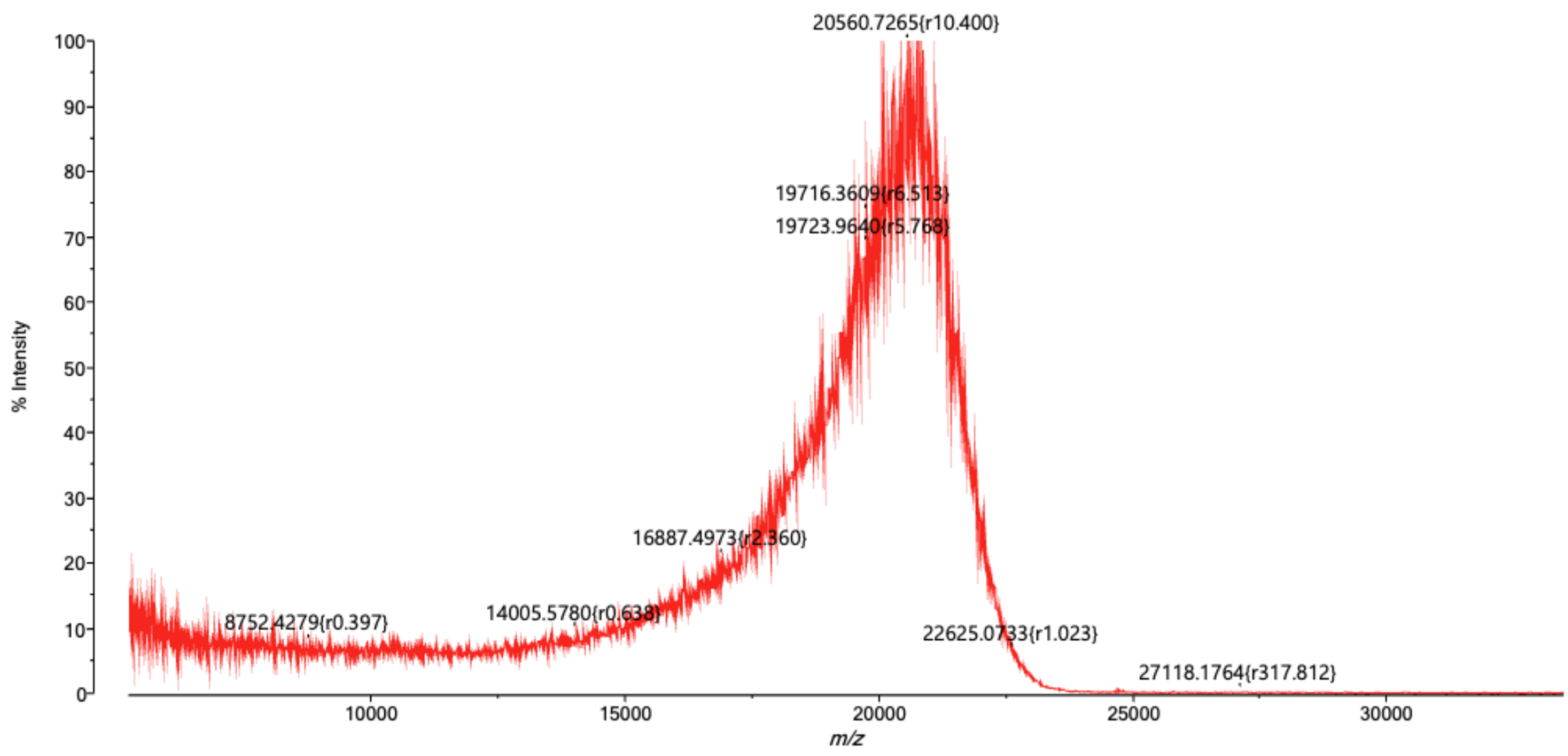

Figure S2. Intact-mass analysis of purified db-siRNA by MALDI-TOF MS.

The observed dominant peak was compared with the theoretical mass calculated from the designed 65-nt db-siRNA sequence(CUAACCCUAUUCUCCUUCUUCGCCAA AUUCAAGAGAUUUGGCGAAGAAGGAGAAUAGGGUUGGGU, A_{18} C_{13} G_{16} U_{18}, MW≈20,927.54 Da).

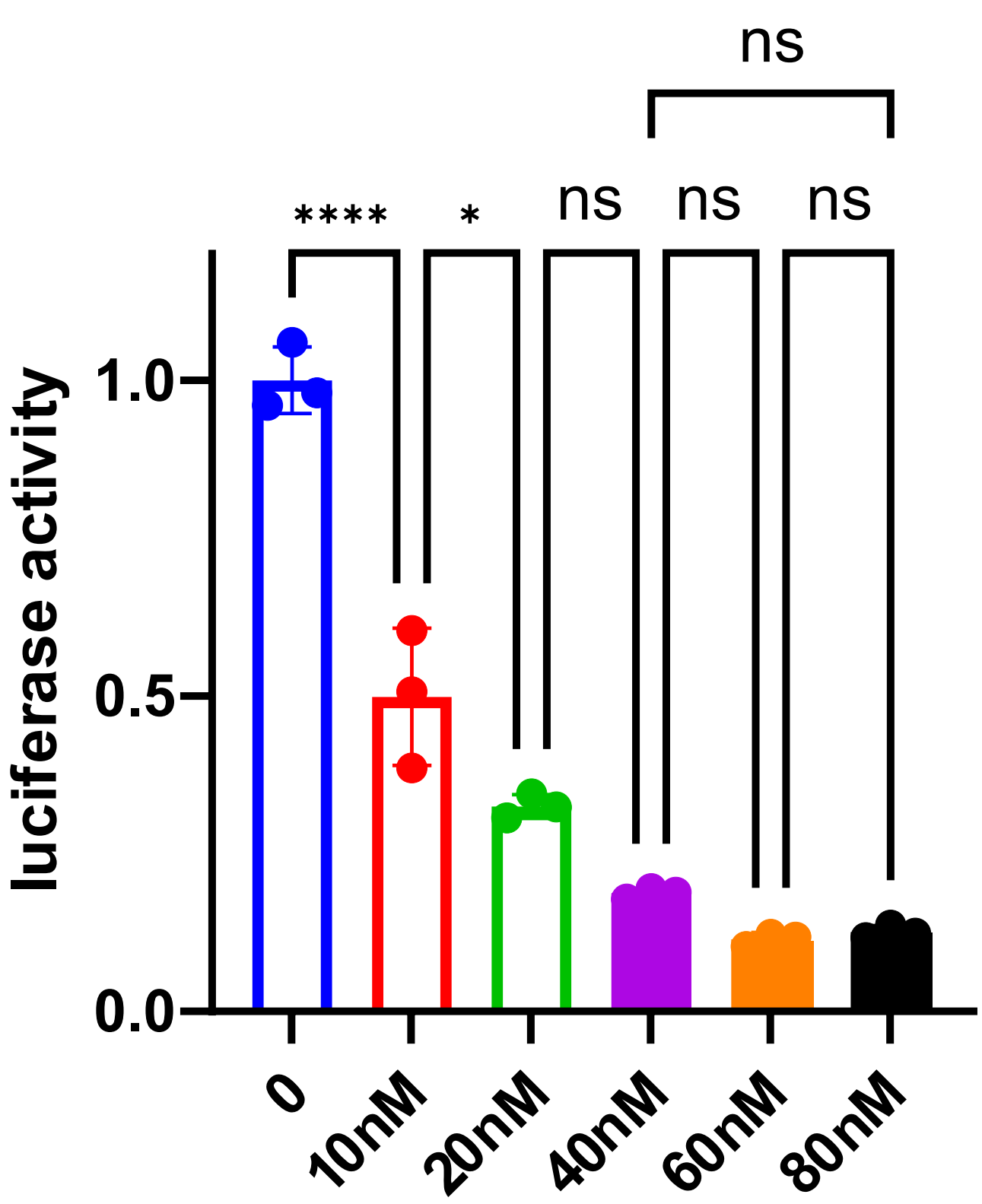

Figure S3. Dose-response analysis of db-siRNA-mediated luciferase silencing.

HEK293T cells were transfected with pcDNA3-Luciferase plasmids and db-siRNA by Lipo8000 at the indicated final concentrations, and luciferase activity was measured 24 h post transfection and normalized to the scrambled siRNA control. Statistical annotations are shown in the graph. Data are shown as mean ± SD; ns, non significant.*, p<0.05; **, p<0.01; ***, p<0.001;_****p<0.0001.

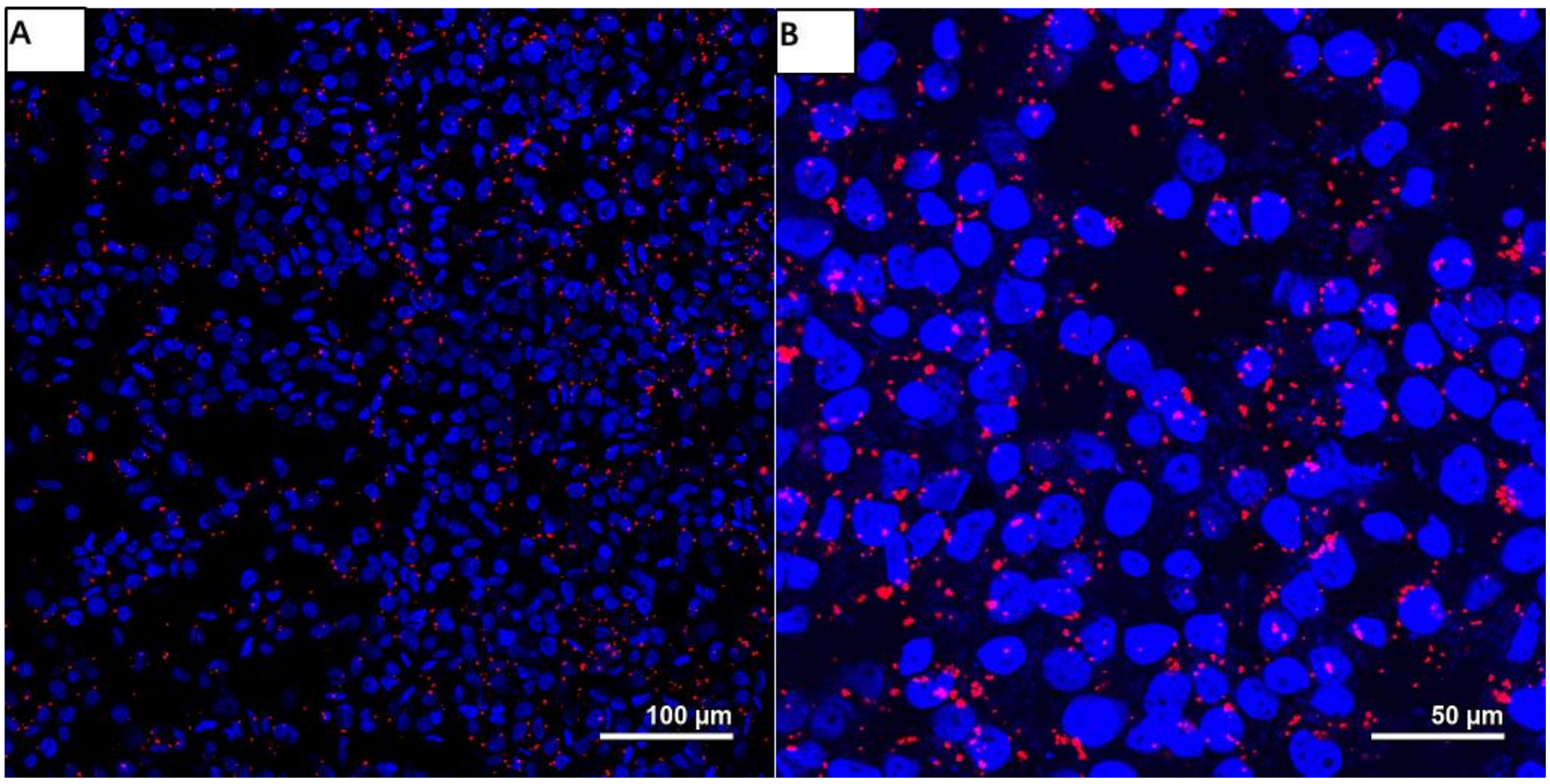

Figure S4. Cellular uptake of Cy5-labeled db-siRNA.

Cy5-labeled db-siRNA was transfected into HEK293T cells using Lipo8000. Image-based cell counting indicated that approximately 80% of cells contained detectable Cy5 signal. Scale bars, 100 μm (A) and 50 μm (B).

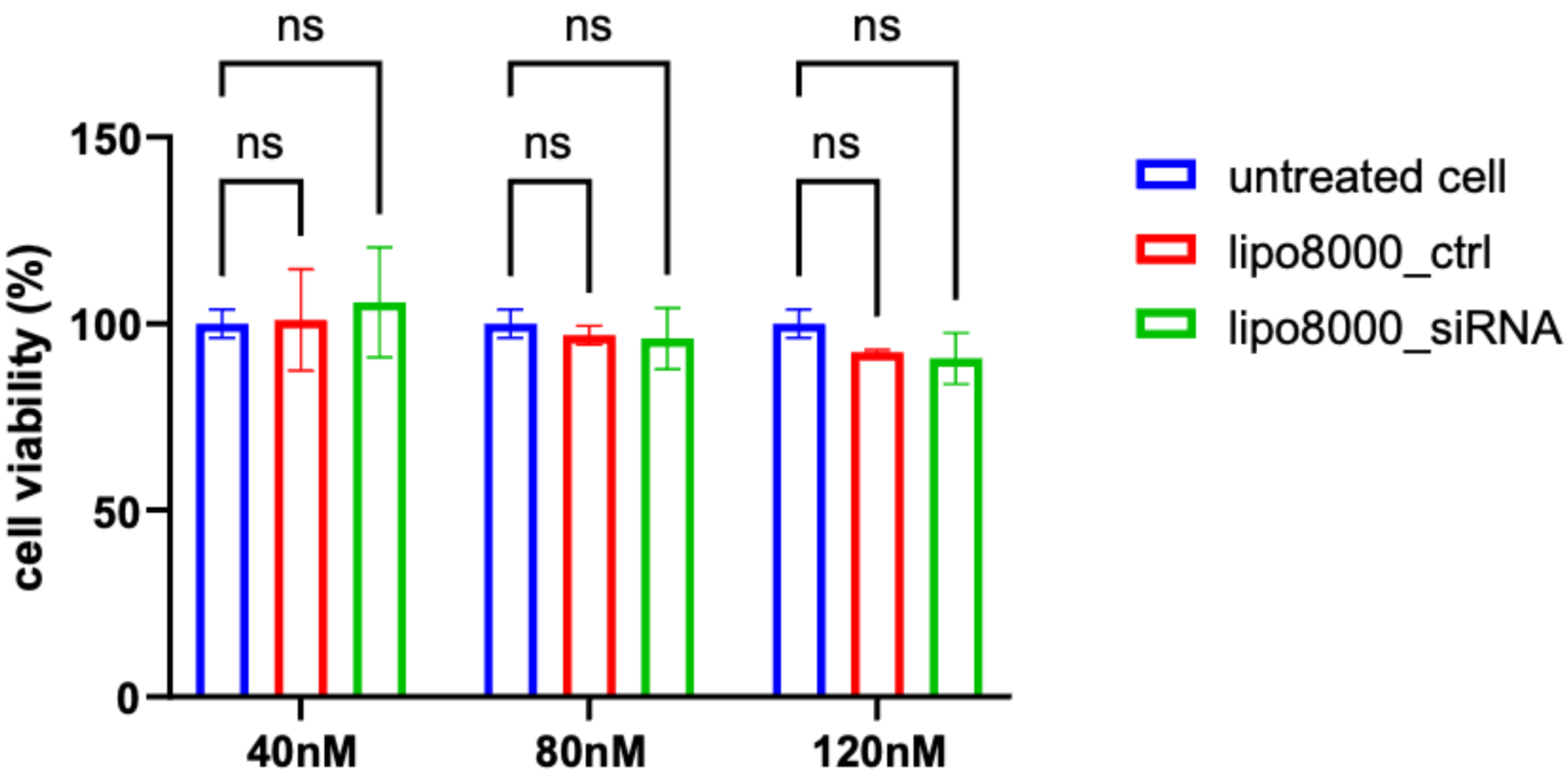

Figure S5. Cell viability after db-siRNA transfection.

HEK293T cells were treated with Lipo8000 alone or db-siRNA/Lipo8000 complexes at the indicated concentrations for 24 h. Viability was measured by CCK-8 assay and normalized to untreated cells. Data are shown as mean ± SD; ns, non significant.

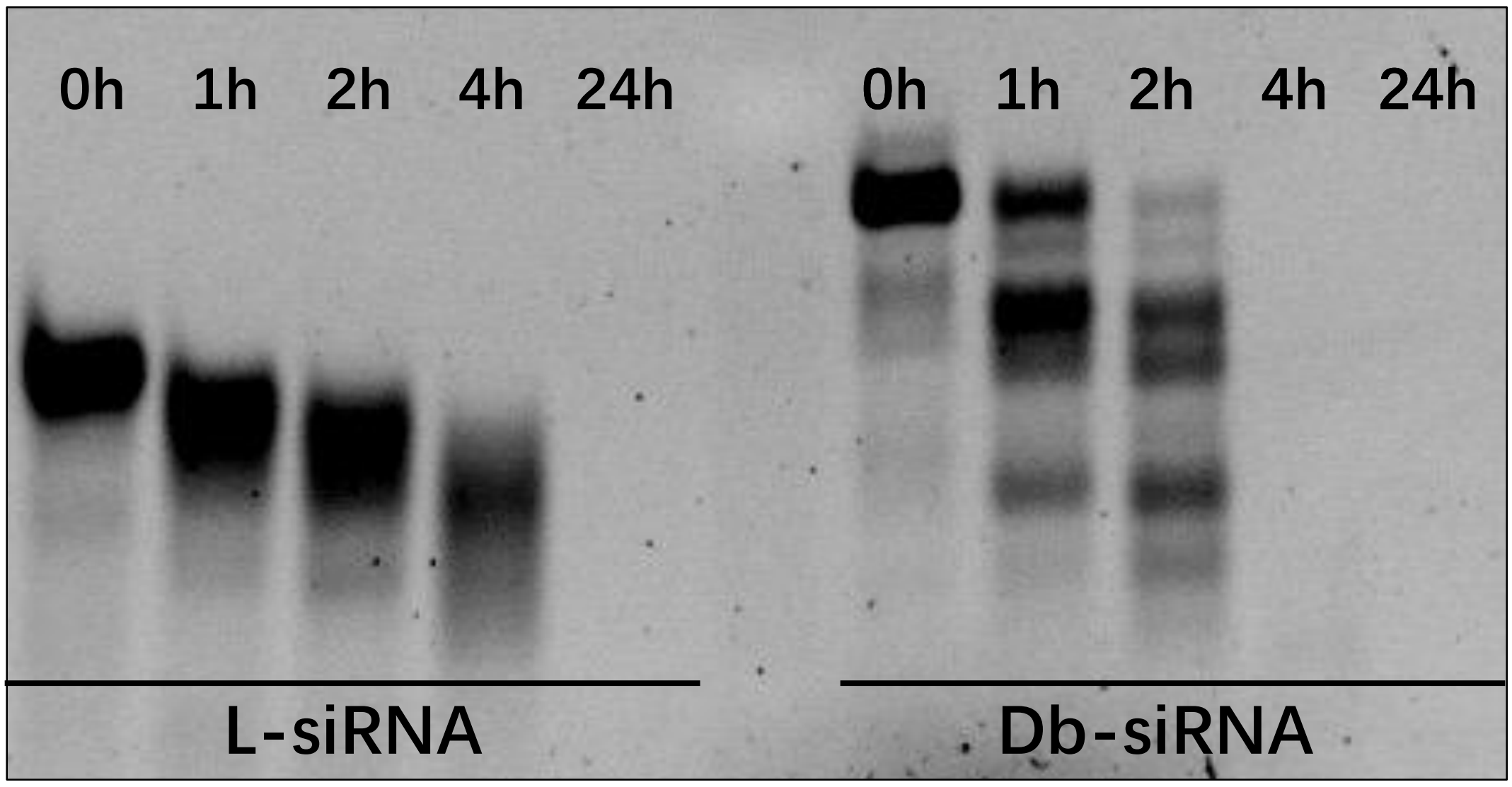

Figure S6. Serum stability of db-siRNA and linear siRNA in 20% FBS.

Samples were collected at the indicated time points and analyzed by native PAGE. L-siRNA, linear siRNA; db-siRNA, dumbbell-shaped siRNA.

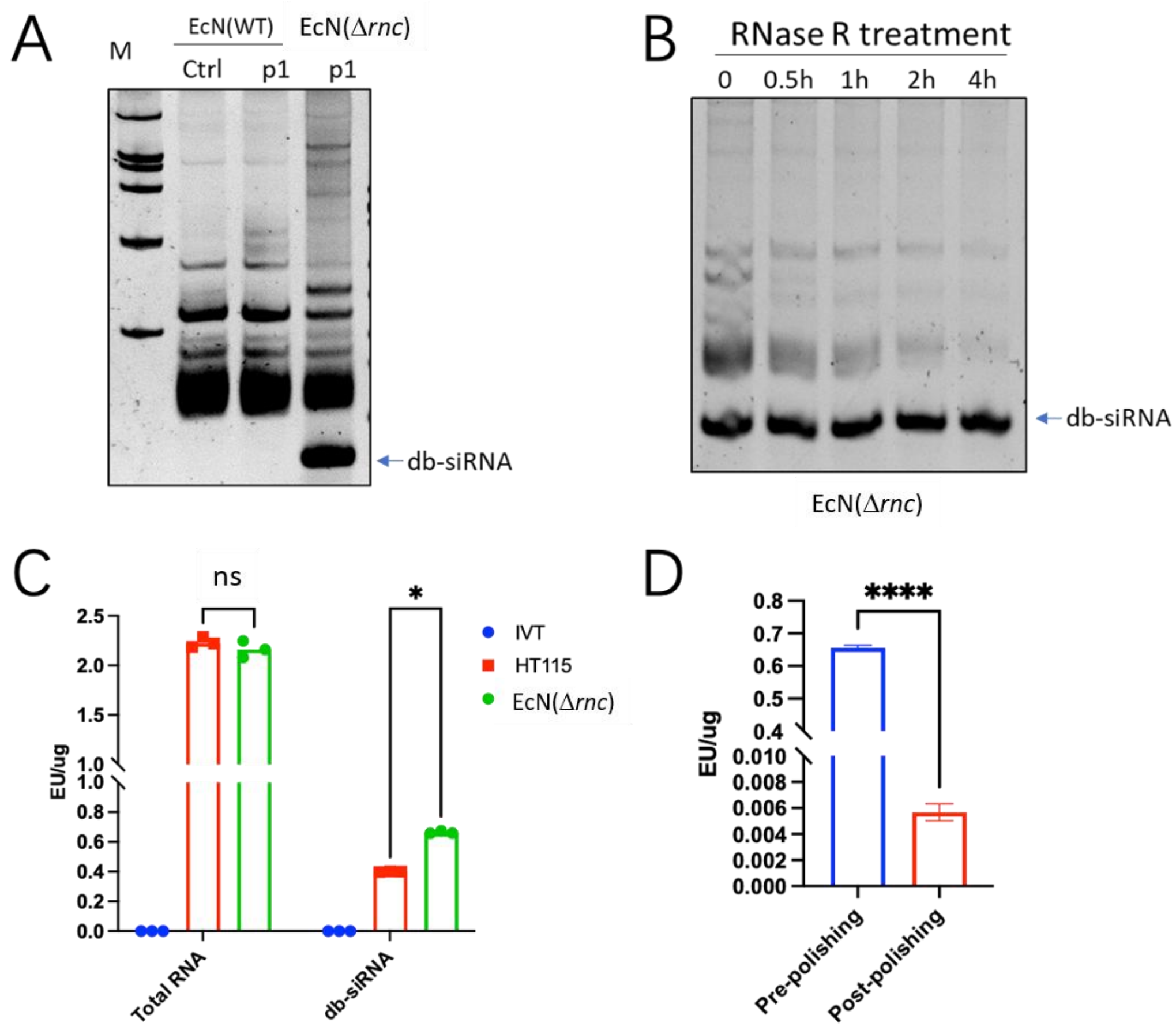

Figure S7. EcN-based db-siRNA production, immunogenicity evaluation, and endotoxin removal.

(A) Native PAGE gel electrophoresis for total RNAs isolated from the EcN. Ctrl, no plasmid transformation; p1, pET28a-db-siRNA transformed.

(B) Native PAGE gel electrophoresis for total RNAs isolated from the EcN *ΔRnc* strain 0.5h, 1h, 2h and 4h post digestion by RNase R.

(C) Quantitative analysis of endotoxin across various manufacturing platforms. Comparative profiling of residual endotoxin contamination levels within crude total RNA extracts and pure db-siRNAs generated via *in vitro* transcription (IVT,

blue), *E. coli* HT115 fermentation (orange), and EcN(Δ*rnc*) fermentation (green). *$p<0.05$; nd denotes not detectable.

(D) Endotoxin removal for db-siRNAs from EcN generation using 1% Triton X-114 extraction. Triton X-114 phase separation reduced residual endotoxin to approximately 0.005 EU/μg RNA. Data are shown as mean ± SD; ns, non significant; *, $p<0.05$; **, $p<0.01$; ***, $p<0.001$;_****$p<0.0001$.

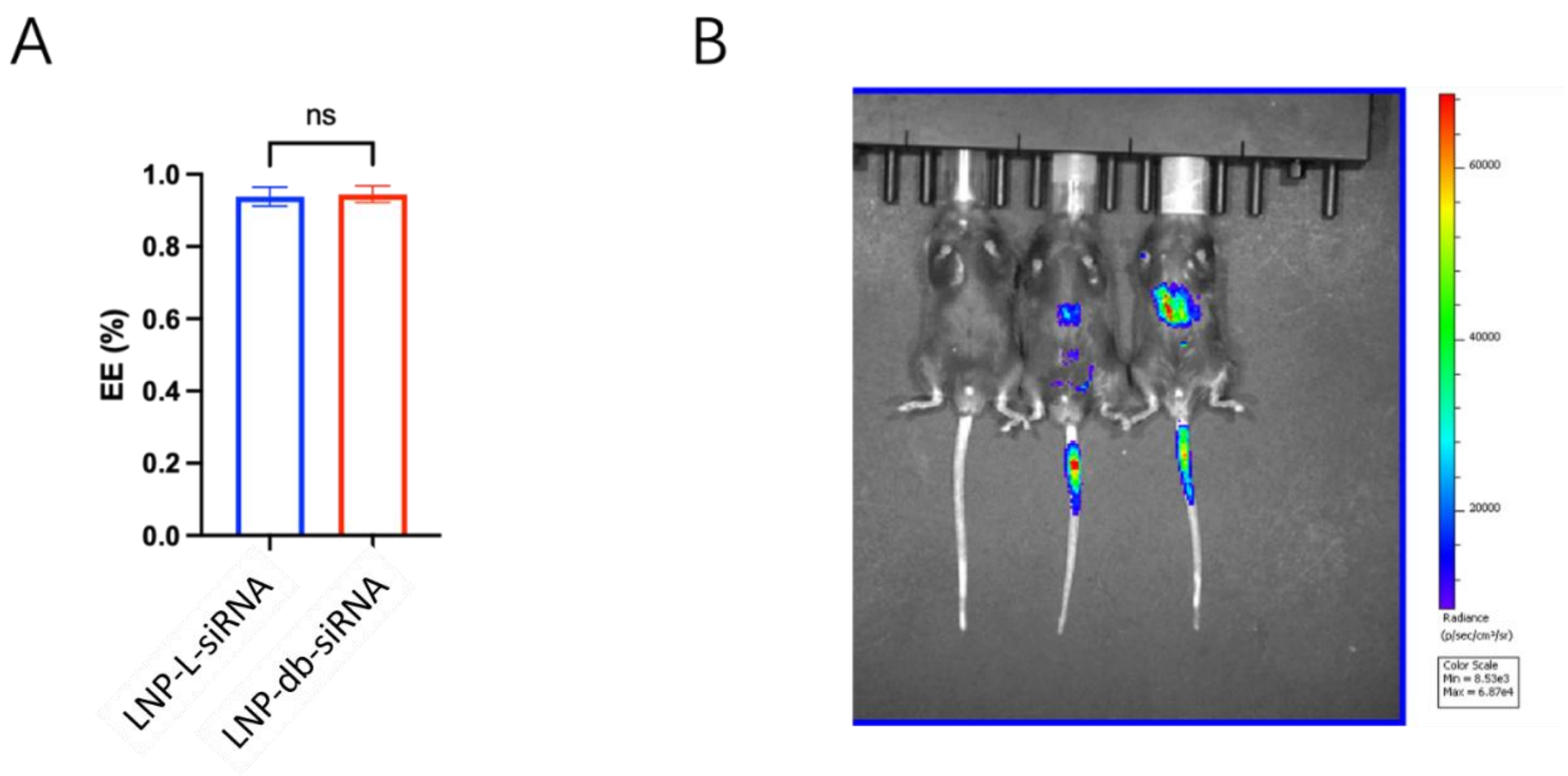

Figure S8. Db-siRNA encapsulation efficiency analysis and LNP hepatic delivery.

(A) Comparative encapsulation efficiency of linear siRNA and db-siRNA determined by RiboGreen assay.

(B) Hepatic delivery of LNP-FLuc mRNA in mice. IVIS imaging of mice 12 h post intravenous injection of LNP-FLuc mRNA at dose of 0, 5, and 10 μg from left to right. Data are shown as mean ± SD; ns, non significant.

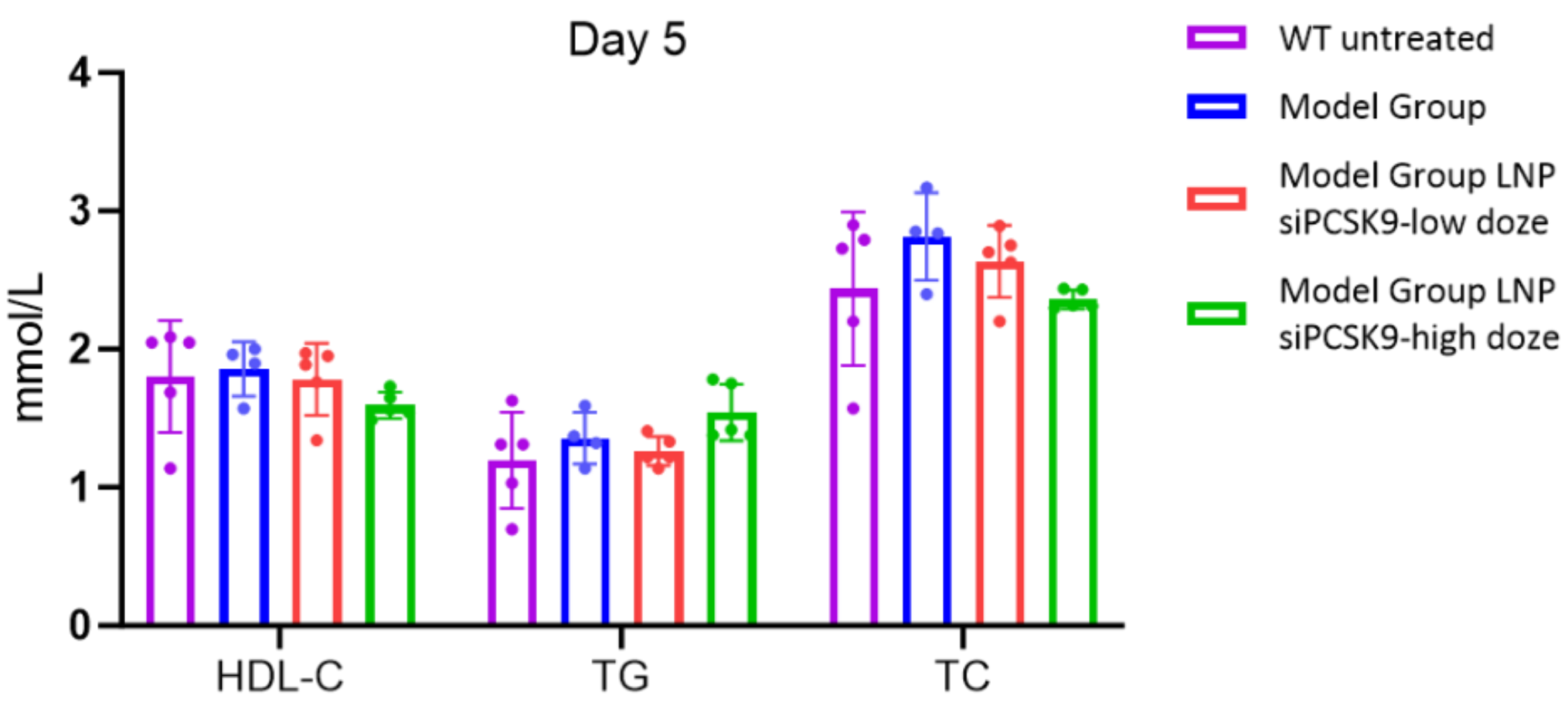

Figure S9. LNP-encapsulated db-siRNAs knockdown PCSK9 in mice.

HDL-C, TG and TC levels in serum samples from hyperlipidemia mice 5 days post injection of LNP-db-siPCSK9-3 RNAs.

Table S1

The DNA sequences of siRNA, oligos for PCR and EcgRNA for genome editing were listed in the separate supplemental table S1 as an Excel file.
